# Sex- and ethnic differences in the cross-sectional association between sleep regularity and obesity among US adults, NHANES 2011-2014

**DOI:** 10.1177/07487304251391267

**Published:** 2025-12-18

**Authors:** Jürgen Degenfellner, Eva Schernhammer, Susanne Strohmaier

**Affiliations:** 1 Department of Epidemiology, Center for Public Health, Medical University of Vienna, Kinderspitalgasse 15, 1090 Vienna, Austria; 2 Institute of Physiotherapy, ZHAW School of Health Sciences, Katharina-Sulzer-Platz 9, Winterthur, Switzerland; 3 Channing Division of Network Medicine, Brigham and Women’s Hospital and Harvard Medical School, 181 Longwood Ave, Boston MA 02115, USA; 4 Department of Epidemiology, Harvard T.H. Chan School of Public Health, Huntington Ave, Boston MA 02115, USA

**Keywords:** sleep regularity, weight, body mass index, obesity, NHANES

## Abstract

**Background:**

Obesity is a major public health concern, with disparities across racial and sex groups. While sleep duration has been extensively studied in relation to obesity, the role of sleep regularity remains less explored.

**Methods:**

In two nationally representative samples of US adults in the National Health and Nutrition Examination Survey (NHANES 2011/12 & 2013/14, n=7,085), we investigated the cross-sectional association between a sleep regularity index (SRI) derived from accelerometer data and obesity measures. Body Mass Index (BMI), waist circumference (WC), body roundness index (BRI), total fat mass, sagittal abdominal diameter (SAD), sagittal abdominal diameter to height ratio (SADHtR), fat mass index (FMI), lipid accumulation product (LAP), and visceral adiposity index (VAI) were derived from NHANES body measures. Multivariable-adjusted regression models were used to estimate multiplication factors (MF) and 95% CIs comparing mean BMI across quintiles of SRI and to test for effect modification by sex and ethnicity.

**Results:**

Higher SRI was associated with significantly lower BMI (MF SRI_Q5vs.Q1_: 0.92; 95%CI, 0.91-0.94; P_trend_<0.001), translating into 8% lower BMI among those with most versus least regular sleep. This association was more pronounced among women than men (MF SRI_Q5vs.Q1_ women: 0.92; 95% CI, 0.90-0.95; men: 0.98; 95% CI, 0.96-1.00), with strongest effects in non-Hispanic white and other/multi-racial women (P_interaction_<0.001). Similar inverse associations were observed for all other obesity measures.

**Conclusion:**

Sleep regularity, measured by the SRI, was inversely associated with BMI and any other obesity measures. The observed disparities suggest sleep regularity may contribute differentially to obesity risk by sex and race/ethnicity.

## Introduction

Obesity remains a significant global public health challenge, with rising prevalence rates contributing to increased risk for numerous chronic conditions, including cardiovascular disease, type 2 diabetes, and various cancers (Ahmed & Konje, 2023). In the United States alone, obesity rates among adults have reached more than 40% (CDC, 2025), highlighting the urgent need to understand risk factors contributing to obesity and weight gain.

Among these factors, sleep has emerged as a key area of interest, with growing evidence linking not only sleep duration but also irregular sleep patterns to obesity and related health outcomes (Ogilvie & Patel, 2017). Traditionally, research has largely focused on short sleep duration as a risk factor for obesity (Cappuccio et al., 2008). However, recent studies suggest that irregularity in sleep timing might also play a crucial role (Zuraikat et al., 2020).

According to a consensus statement of the National Sleep Foundation, daily regularity in sleep timing is important for health and performance (Sletten et al., 2023). While it has long been known that night work with its irregular sleep and mealtime patterns contributes to ill health (Kecklund & Axelsson, 2016; Nea et al., 2015), more recently, this notion has also become center and focus for individuals encountering less drastic changes in sleep patterns than night workers. To date, sleep irregularity has been linked to a broad range of adverse health outcomes, including impairments in mental health (Messman et al., 2024), metabolic dysfunction (Zhu et al., 2022) and cardiovascular risk (Huang et al., 2020). Disruptions in sleep patterns can disturb circadian clock synchronization, alter the expression of key metabolic genes, reduce insulin sensitivity, and thereby increase obesity risk (Ansu Baidoo & Knutson, 2023; Noh, 2018). In addition, changes in the timing of meal intake – often accompanying irregular sleep – have more recently been identified as an independent risk factor for obesity (Chellappa et al., 2025).

Several metrics have been suggested to measure consistency in individuals’ sleep-wake schedules (Zhang & Qin, 2023), including traditional overall measures such as the standard deviation (e.g., applied to sleep duration), inter-daily stability (Witting et al., 1990), and social jetlag (Wittmann et al., 2006), as well as consecutive measures like the composite phase deviation (Fischer et al., 2016), which was introduced in the context of shift work. Additionally, Phillips et al. (Phillips et al., 2017) recently introduced the sleep regularity index (SRI), which assesses the probability that a person is in the same sleep or wake state at two points 24 hours apart, with higher scores indicating more consistent sleep patterns.

Previous studies (Zhang et al. (Zhang & Qin, 2023), Supplementary Figure 3S) have examined various sleep regularity metrics—including standard deviation of sleep duration, inter-daily stability (ISI), the sleep regularity index (SRI), and social jet lag (SJL)—in relation to BMI. Most studies found that greater variability in sleep timing was associated with higher BMI, though findings for ISI and SJL were mixed.

So far, only two studies have specifically focused on the association between SRI and BMI (Lunsford-Avery et al., 2018; Wong et al., 2022). However, both were conducted in narrowly defined populations (younger or older adults) and did not account for ethnic and sex-based differences. To address this gap, our study uses nationally representative data from U.S. adults to examine potential differences by sex and ethnicity. This approach is important because understanding how sleep regularity relates to obesity across diverse demographic groups is critical for informing tailored public health strategies. Given the reported variation in sleep regularity and obesity prevalence across race and sex (Chung et al., 2021; Huang & Redline, 2019; Petersen et al., 2019), the relationship between sleep regularity and obesity might also vary by these demographic factors. By exploring these differences, our study seeks to contribute to the growing body of evidence on the role of sleep regularity in the aetiology of obesity.

## Methods

### Study population

This study utilized data from NHANES participants who were 20 years and older from the two survey cycles 2011–2012 and 2013–2014, specifically selected due to the availability of 24-hour accelerometer data, leaving 7,085 NHANES participants to constitute our study sample. NHANES implemented a multistage probability sampling method to create a representative, weighted sample of the U.S. population (Chen et al., 2018). Ethical approval for NHANES protocols was granted by the National Center for Health Statistics Research Ethics Review Board, with informed consent obtained from all participants. Data were accessed in March 2024 from the publicly available NHANES 2011–2014 datasets via the CDC website. The data are de-identified public-use files. Authors had no access to personally identifying information at any point before, during, or after data analysis.

### Accelerometer data collection and preprocessing

All participants in both the 2011–2012 and 2013-2014 NHANES cycles were asked to continuously wear an accelerometer (ActiGraph Model GT3X+, ActiGraph, Pensacola, FL), day and night for 7 consecutive days. The accelerometer, which was ideally worn on the non-dominant wrist, recorded raw signals on the x-, y-, and z-axes at a frequency of 80 Hz (Health & Survey, 2024a, 2024b). These signals were processed, flagged, and summarized at the minute level. The resulting data were released by NHANES in November 2020. The minute-level summary data (PAXMIN) were specified in Monitor-Independent Movement Summary (MIMS) units, a universal summary metric that is non-proprietary, open-source, and device-independent, developed by researchers at Northeastern University (John et al., 2019).

**Figure 1:**
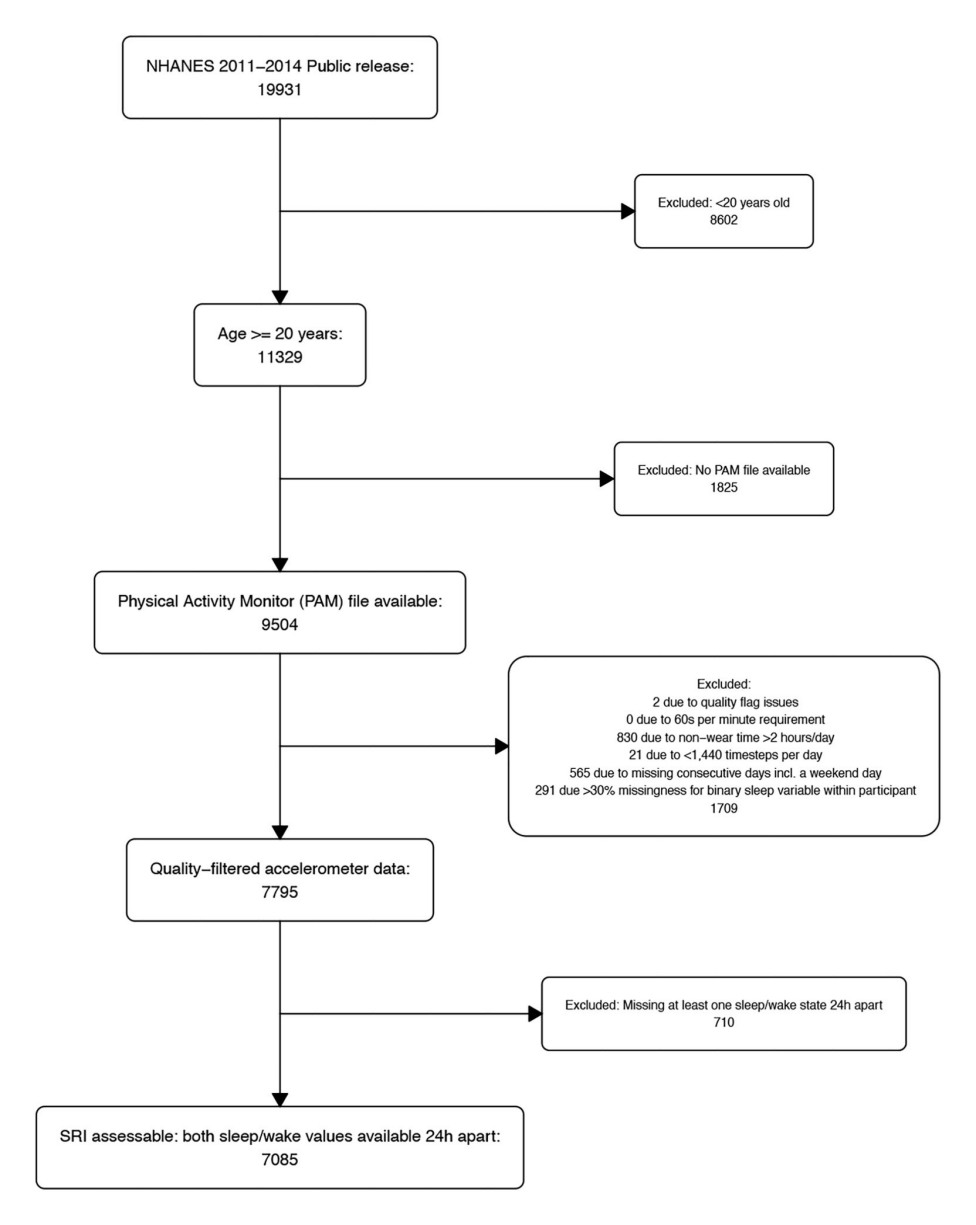
Inclusion of participants from the NHANES 2011–2014 dataset. The final sample includes 7,085 participants. The study population consists of participants who are at least 20 years old, a physical activity monitor file (PAM) is available, have passed quality filters for accelerometer data and where the sleep regularity index was defined. A stratified version of this flowchart, separating NHANES 2011-12 and 2013-14, is provided in Supplementary figure 10S.

The accelerometer data were filtered to ensure quality. Only data without quality flags and with exactly 60 seconds per minute were included. Less than 2 hours of non-wear time per day were required. Each day was required to consist of 1,440 timesteps (60 timesteps per hour for 24 hours). Each participant was required to have at least three consecutive days of data, including either a Saturday or Sunday. Participants with missingness of more than 30% for the binary sleep variable (PAXPREDM = NA/non-wear/unknown) across the observation period were also excluded. Figure 1 displays the inclusion and exclusion process used to derive the analytic sample. A version stratified by survey year is provided in Supplementary figure 1S.

### SRI (exposure)

Participants activity status during each minute was grouped into four categories (wake/sleep/non-wear/unknown) using an open-source machine learning algorithm (using the variable PAXPREDM (CDC; CDC)). We recoded the variable PAXPREDM to define: 1 = ‘sleep’; 0 = ‘wake’ and missing (NA) if PAXPREDM = ‘non-wear’ or ‘unknown’. See Supplementary Figure 2S for examples of visualizations of the binary sleep variable. For the SRI calculation we used the original formula provided by Philips et al. (Phillips et al., 2017), which can be found in the Supplementary material of Windred et al. (Windred et al., 2021):

$$SRI=-100+200(1-\frac{1}{N_{v}}\sum_{i=1}^{Nv}|s_{i}-s_{i+C}|)$$

Sleep-wake states are represented by *s*_*i*_ = 0 for wake, *s*_*i*_ = 1 for sleep, and *s*_*i*_ = NA represents excluded epochs. Number of valid epoch-by-epoch comparisons is represented by *N*_*v*_, which includes all comparisons where *s*_*i*_ ≠ NA and *s*_*i*+*c*_ ≠ NA. Where *s*_*i*_ = NA or *s*_*i*+*c*_ = NA, |*s*_*i*_ – *s*_*i*+*c*_| = 0. In our case, c = 1,440 minutes.

The theoretical range of the SRI is -100 to 100, although for most real sleep/wake patterns SRI values fall into the range 0 to 100 (Fischer et al., 2021). To address negative values in our sample, we applied a winsorization approach using simulated random noise. We first simulated the expected SRI distribution under random sleep patterns, assuming 1,440 minutes per day over two days, to establish reasonable cutoffs. A Monte Carlo simulation of 100,000 SRI values based on random sleep yielded a 99% quantile range of approximately ±6.8. Thus, SRI values below -6.8 were replaced with random noise drawn from a normal distribution with the mean and standard deviation of the before simulated random SRI values. This replacement was applied only for SRI values below -6.8 since an SRI close to zero reflects random sleep, while positive values indicate more regular patterns. By restricting winsorization to the negative direction, we preserved the interpretation of zero as a baseline for random sleep. Eight SRI values (out of a total of 7,085) were winsorized in this fashion.

### Body mass index (BMI) and other obesity measures

All anthropometric measurements, including weight, height, waist circumference, and sagittal abdominal diameter, were obtained by trained health technicians during standardized examination visits at mobile examination centers, following NHANES protocols (CDC; CDC). BMI was calculated as weight in kilograms divided by height in meters squared, and then rounded to one decimal place (CDC; CDC). To limit the influence of extreme values, we applied winsorization to the BMI. Values below the 1st percentile (18.01 kg/m^2^) and above the 99th percentile (51.80 kg/m^2^) were capped at these respective thresholds and set to the respective nearest quantiles (see Supplementary figure 3S).

While BMI remains the most commonly used, widely accepted, and practical measure of obesity in adults, its interpretation may be limited, particularly in ethnically diverse populations (Adab et al., 2018). To address these limitations, we incorporated additional obesity measures into our analysis. These included waist circumference (WC) in cm, which captures central obesity, and the waist-to-height ratio (WHtR), which adjusts WC for stature, is dimensionless and defined as waist circumference in cm divided by body height in cm. We also included indices that capture aspects of body shape and fat distribution beyond BMI. ‘A Body Shape Index’ (ABSI) incorporates waist circumference adjusted for height and BMI, thereby quantifying abdominal adiposity independently of overall body size. Higher ABSI values indicate greater central fat concentration and have been associated with increased risk of premature mortality (Krakauer & Krakauer, 2012). The body roundness index (BRI) is derived geometrically from waist circumference and height, treating the body cross-section as an ellipse to estimate roundness. Higher BRI values reflect a rounder body shape and greater visceral adiposity (Thomas et al., 2013). The Visceral Adiposity Index (VAI) is calculated using sex-specific equations that combine waist circumference, BMI, triglycerides, and HDL cholesterol. It serves as a surrogate marker of visceral adipose distribution and function, with higher values indicating elevated risk of insulin resistance, metabolic syndrome, and cardiovascular disease (Amato et al., 2010). Formulae for ABSI, BRI and VAI can be found in Koyama (Koyama, 2023). To adjust fat mass for body size, we utilized the fat mass index (FMI), defined as fat mass in kg divided by height in m squared, and to reflect abdominal fat distribution, we included the sagittal abdominal diameter (SAD) in cm and the sagittal abdominal diameter to height ratio (SADHtR) (Zhang et al., 2022). The lipid accumulation product (LAP) was derived from triglyceride levels and waist circumference (Koyama, 2023). WC, total fat mass in kg, total fat percentage and SAD were winsorized in the same manner as BMI.

### Covariables

Information on ethnicity, occupational category, education, annual household income, smoking status, alcohol consumption, marital status, depression, activity levels, vitamin D levels, total caloric intake, timing of last meal and eating window were available. Levels of categorical variables can be found in Figure 1 and detailed information on covariables is provided in the Supplementary material. The hypothesized relationships on potential confounders between SRI and BMI were illustrated to provide a clear conceptual framework (see Supplementary figure 4S).

### Statistical Analysis

We calculated medians and quartiles for continuous variables (including all obesity measures), and absolute numbers and percentages for categorical variables, stratifying by quintiles of the SRI. We additionally used Pearson correlation coefficients to describe the associations between the different adiposity measures. BMI was right-skewed and therefore was log-transformed to improve model fit. We fit four regression models of SRI with log(BMI). Model 1 included age and sex; model 2 additionally included all other covariables from Table 1 (race/ethnicity, education level, household income, occupational category, marital status, alcohol consumption, smoking status, vitamin D level, total caloric intake, PHQ-9 depression score and activity level); models 3&4 further included interaction terms for sex and race/ethnicity (for additional information see also Suppl. Fig. 5S&6S). Based on these models effect modification for sex and race/ethnicity was tested using the Rao-Scott likelihood ratio test. Since actigraphy data were available for both the 2011-2012 and 2013-2014 cycles, 4-year MEC survey weights were calculated following CDC guidelines (NHANES, 2024) and used for all regression models. We used the R package survey to appropriately consider weights in the models (Lumley, 2004).

Due to the log transformation, we present multiplication factors (i.e. percentage changes) and bootstrap confidence intervals for average BMI per median of SRI quintiles overall and stratified by sex and race/ethnicity. These factors are derived by exponentiating corresponding linear combinations of regression coefficients. To aid model interpretation we provide estimations and bootstrap confidence intervals for BMI across the full range of SRI, based on model 4 and stratified by sex and race/ethnicity. Covariable levels were set to survey-weighted medians for skewed continuous variables, while survey-weighted means were applied to symmetric continuous variables. For categorical variables, the most common category (mode) was used. Additionally, we randomly drew 200,000 covariable values and applied model 4 to estimate corresponding BMI realisations.

In addition to BMI, we also fit model 4 to the other obesity measures, again applying a log-transformation where appropriate. To address potential confounding by meal timing, we additionally added available covariables to model 4 - difference in timing of the last meal and difference in eating window between weekdays and weekend - in the subset of individuals providing that information.

### Handling of missing values in the covariables

Missingness in the analytic data set before imputation ranged from 0% to a maximum of 24% (for alcohol consumption; see Supplementary Figure 7S). Missing values were imputed using k Nearest Neighbour imputation using the R package VIM (Kowarik & Templ, 2016). Total fat mass, FMI, and total percent fat had 49% missing data and models were fit on the non-imputed dataset. The meal timing sensitivity analysis was performed on complete cases due to 38% missing values in the relevant covariables.

## Results

Among the 7,085 NHANES participants that constituted our study sample, (survey weighted) mean SRI was 61.3 and 38.6% of participants in our sample were obese. Characteristics of the study population overall (n=7,085) and stratified by SRI quintiles are presented in Figure 1. The median age of participants was 52 (interquartile range 36 to 65) years, 53% of the total sample were female. The survey-weighted (raw) comparison between women and men revealed an SRI difference of 2.4 points, with females exhibiting a higher median SRI (62.22) than males (59.86). The largest racial group was Non-Hispanic White (41%), followed by Non-Hispanic Black (23%). Across SRI quintiles, the proportion of Non-Hispanic Black participants declined from 33% in quintile 1 (lowest sleep regularity) to 13% in quintile 5 (highest sleep regularity). In contrast, the proportion of individuals with the highest level of education increased from 17% in Q1 to 35% in Q5, while the proportion of low-income participants (< $20,000) was halved. Employment status varied markedly, with working participants being more than twice as prevalent in Q5 than in Q1 (70% vs. 32%). Similarly, the proportion of married individuals increased across quintiles. Health-related behaviours and lifestyle factors also exhibited clear patterns. Heavy drinkers and smokers were twice as prevalent in Q1 compared to Q5, while total caloric intake was approximately 10% higher in Q5 than in Q1. Additionally, depression scores showed a decreasing trend, while physical activity levels increased progressively from Q1 to Q5. Similar trends were observed for each survey year separately (see Supplementary Figures 1S and 2S).

Table 2 provides a descriptive overview of the considered obesity measures across quintiles of SRI. Our primary outcome, BMI, showed a slight decrease in median values from 29 to 27 kg/m^2^ from the first to the fifth SRI quintile. Most other obesity measures exhibited a slight downward trend across quintiles as well, with the exception of percent body fat where the tendency is less consistent.

Results from regression analyses indicated that higher SRI values were significantly associated with lower BMI in the sex- and age-adjusted model as well in the multivariable adjusted model (model 1 and 2 in Table 3).

Investigation of sex and race/ethnicity specific estimates revealed that the inverse association between SRI and BMI seemed to stronger among women (women: MF for SRI, top (Q5) versus bottom (Q1) quintile: 0.92, 95%CI 0.90-0.95; men: 0.98, 95% CI 0.96-1.00), with MFs, comparing Q5 vs. Q1, ranging from 0.87 (95%CI, 0.80-0.95) for other/multi-racial women to 0.98 (95%CI, 0.94-1.02) for Mexican American women. Among males, the tendency of this relationship was less consistent. For Non-Hispanic White males, we observed multiplication factors below one across all quintiles (e.g., comparing Q5 to Q1: 0.97, 95%CI 0.94-0.998), while the factors for Mexican American men were consistently above 1 (e.g., comparing Q5 to Q1: 1.05, 95%CI, 1.00-1.09). The association between SRI and BMI varied significantly by sex and race/ethnicity (overall P_int_ = 4.5e-05), with both interactions evident when tested separately.

**Figure 2:**
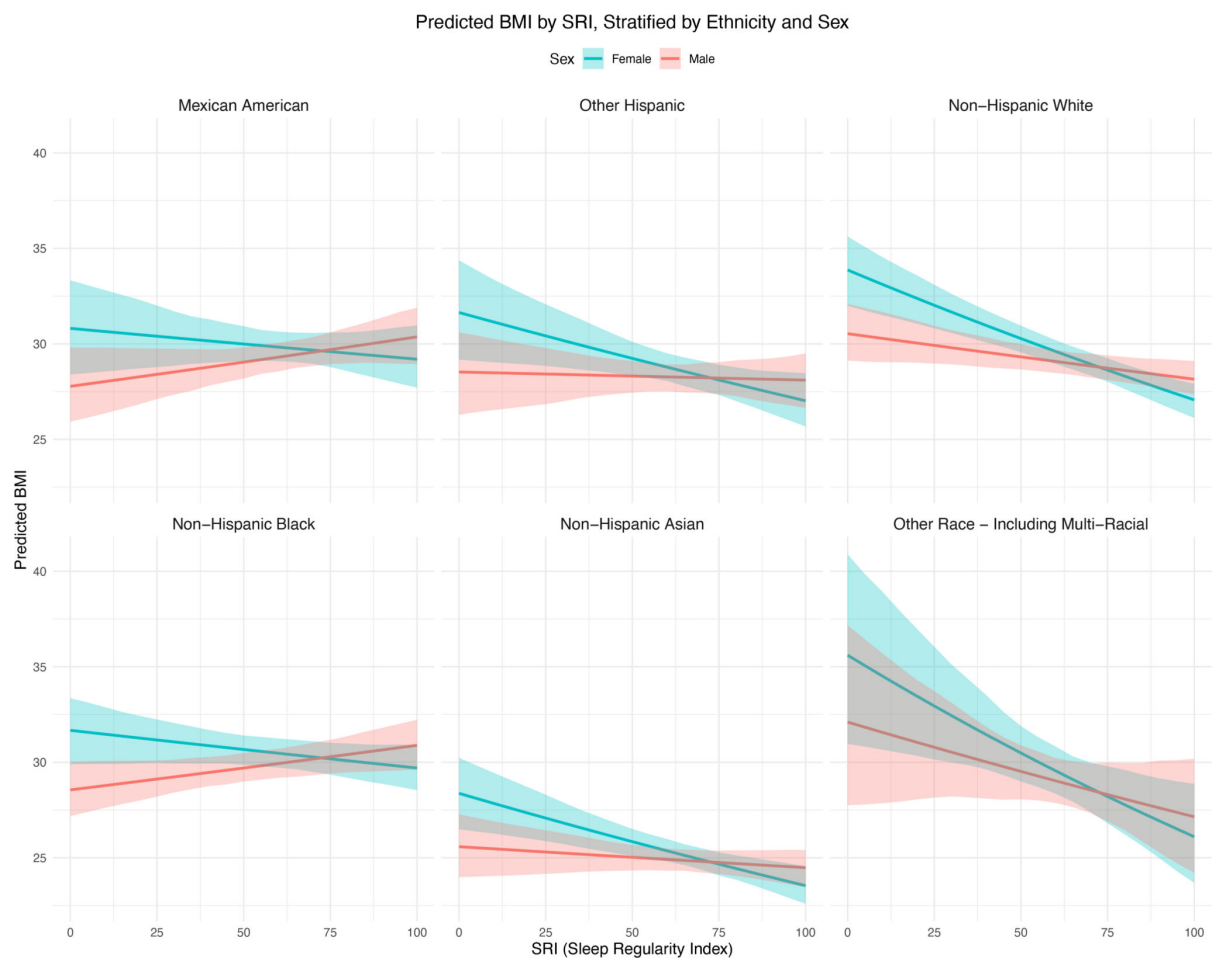
Fully adjusted model predictions from model 4 for BMI across the full SRI range (0-100) with 95% (bootstrap) confidence bands stratified by sex/gender and race/ethnicity.

Figure 2 shows the BMI values across the whole SRI range (0-100) based on model 4. Corresponding to Figure 2, the decline in BMI was most pronounced among other/multi-racial women (approximately 10 BMI units across the entire SRI range), whereas for Mexican American women, in accordance with model 4, the trend was compatible with no effect. Among males, BMI remained relatively stable across SRI levels, suggesting little to no association between SRI and BMI. Considering a wider range of possible covariable combinations, we observed qualitatively identical trends but wider confidence intervals (see Supplementary Figure 8S). In the subset with available with meal timing related variables, we obtained qualitatively similar results which did not materially alter the main findings.

BMI was highly correlated with WC, BRI, WHtR, total fat mass (in kg), SAD, SADHtR and FMI (each r≥0.89) (see Supplementary Figure 9S). Extrapolations across the SRI range were qualitatively similar (see Supplementary Figure 10S). Total percent body fat showed no association with SRI although it correlated moderately with BMI (r = 0.57). Predictions across the SRI range for LAP (r=0.51) and VAI (r=0.23) were qualitatively similar to those of BMI. In contrast, ABSI was uncorrelated with BMI (r=-0.03) and exhibited distinct patterns, showing consistent trends for both sexes.

## Discussion

In this study, we examined the association between sleep regularity and multiple adiposity indices in a large, nationally representative cross-sectional sample of US adults. Our findings suggest that greater sleep regularity is associated with lower levels of BMI, as well as several other measures of adiposity, and that the strength and direction of these associations varied by sex and race/ethnicity. The most pronounced trends were observed for Non-Hispanic White women, suggesting that the full range of sleep regularity observed in this group can lead to differences of 10 units in BMI. Interestingly, meal timing did not appear to impact these associations.

In our study sample 38.6% of participants were obese, which is roughly in line with the nationally representative proportion in the US of 41.9% (Stierman et al., 2021). Also, women exhibited a modestly higher median SRI than men in our sample, which is consistent with findings from Windred et al. (Windred et al., 2021), who reported a slightly smaller difference of 1.2 points between sexes.

Only a limited number of studies have previously examined the association between SRI and BMI. Wong et al. (Wong et al., 2022) reported a negative association between SRI and BMI (β = -0.06) in a sample of college students, adjusting for total sleep time and sex. However, their analysis is not directly comparable to ours, as it did not account for stratification by race/ethnicity, and the mean age of 18.7 years fell below the inclusion threshold of our study. Reportedly, 48% of participants in their sample were non-White, which is lower compared to our sample (59% non-White). They report no mean SRI difference with respect to sex (Table 3 in (Wong et al., 2022)). Lunsford-Avery et al. (Lunsford-Avery et al., 2018) reported a negative correlation between SRI and BMI (r=-0.139) in a sample of older adults from the Multi-Ethnic Study of Atherosclerosis (MESA) (mean age: 68.7 years). However, these findings were not stratified by sex or race/ethnicity, and the mean age was substantially higher compared to our study population (68 vs. 52 years). Nonetheless, the correlation for SRI and BMI in our sample (r = -0.12; 95% CI: -0.14 to -0.10) aligns closely with these and other findings in depressed or adolescent populations (Glasgow et al., 2022; Pye et al., 2021).

SRI is one of several metrics used to assess sleep regularity, and its relationship to BMI should be interpreted in the context of other measures that capture different aspects of sleep variability. Especially, studies using the standard deviation of sleep duration as a measure of sleep regularity have also consistently reported an association between greater sleep irregularity and an increased risk of obesity (Zhang & Qin, 2023). For example, Patel et al. (Patel et al., 2014) found that individuals in the highest quartile of sleep duration variability had BMI values that were 1-1.5 kg/m^2^ higher compared to those in the lowest quartile, with this trend observed consistently across both sexes. Ogilvie et al. (Ogilvie et al., 2016) demonstrated that among a racially diverse older adult cohort, higher night-to-night variability in sleep duration was associated with 1-2 units higher BMI and also higher waist circumference, and total body fat. The reported BMI differences are comparable in magnitude to our overall effect.

To the best of our knowledge, most previous studies identifying an association between sleep regularity and obesity did also not examine or report interactions by sex or race/ethnicity (Zhang & Qin, 2023). Patel et al. (Patel et al., 2021) report similar odds ratios for obesity in both sexes for sleep midpoint variability and higher odds ratios in men for sleep duration variability (1.63 vs. 1.22), although men and women were analyzed from two different study samples.

In studies conducted exclusively with female participants, associations of sleep regularity measures and BMI have been of similar magnitude or more pronounced than those observed in our study. For example, Kim et al. (Kim et al., 2015) reported raw correlations between night-to-night variability indices and BMI ranging from r = 0.2 to r = 0.23 in a cohort of 191 older women (aged 80 and above) in Tokyo. Similarly, Bowman et al. (Bowman et al., 2020) found an unadjusted association (β = 0.2) between sleep regularity (measured as the standard deviation of sleep midpoint) and BMI in 221 midlife women (mean age 52.2 years), suggesting a cross-sectional BMI change of 3.6 to 7.2 points for a 1 to 2 standard deviation (SD = 18 min) increase in sleep variability. Additionally, Schreiber et al. (Schreiber & Dautovich, 2019) (n = 132 midlife women, mean age 52.9 years) reported raw correlations between sleep time variability and BMI (r = 0.133), as well as waist circumference (r = 0.164) both in line with our results with respect to magnitude. There were no further studies exclusively conducted in men, or which reported sex specific results.

Sex differences may influence the relationship between sleep regularity and BMI, with variations in metabolism, circadian regulation, and hormonal responses playing a role. Although changes in sleep regularity as measured by the SRI do not necessarily imply circadian misalignment (one could, for instance, awaken frequently during the night at different times from day to day), there is some evidence that circadian misalignment affects energy balance differently in men and women (Qian et al., 2019). There is also growing evidence for sex differences in hedonic eating after acute sleep loss (Benedict et al., 2012; Finlayson & Dalton, 2012; Lok et al., 2024). In shift workers, women are more likely to overeat (Wong et al., 2010) and non-standard working hours may be especially challenging for those with children and caregiving responsibilities, as it can disrupt daily routines and family life (Beermann & Nachreiner, 1995).

With respect to race/ethnicity distribution, the studies from Bowman et al. (Bowman et al., 2020), Ogilvie et al. (Ogilvie et al., 2016), and Taylor et al. (Taylor et al., 2016) are somewhat comparable to ours. All three studies report overall association in the same direction as our analysis but none of these offers race-specific estimates. Similarly, studies with predominantly White (Häusler et al., 2020; Papandreou et al., 2020; Patel et al., 2014; Rosique-Esteban et al., 2018; Schreiber & Dautovich, 2019; Sohail et al., 2015) or Japanese (Kim et al., 2015; Kobayashi et al., 2013) populations also observed associations in the same direction, though direct comparisons of effect sizes are difficult due to differing statistical methods.

While more generally most sleep-obesity research has focused on White populations, some studies with ethnic minorities (primarily Black, Latino, and Asian groups) suggest significant differences in sleep patterns and their association with BMI while other studies did not support racial differences in the relationship of sleep and obesity as summarized in the review by Jackson (Jackson, 2017). Chronic stress, driven by socioeconomic and environmental factors, plays a key role in both sleep disturbances and obesity risk. Many ethnic minorities are disproportionately exposed to psychosocial stressors, including job strain, financial insecurity, racial discrimination, and neighborhood violence. These stressors trigger prolonged activation of the hypothalamic-pituitary-adrenal (HPA) axis, resulting in elevated cortisol levels, which promote fat storage, particularly in the abdominal region. Racial disparities in the sleep-obesity relationship remain understudied, despite the higher obesity risk among racial minorities (Jackson, 2017).

In our analysis, adjustment for a range of demographic, socioeconomic, and lifestyle factors did not markedly change the race-specific results. This implies that the observed racial differences in the association between SRI and BMI persist even after accounting for a broad range of potential confounders. These findings suggests that factors beyond those explicitly included in our models—such as unmeasured cultural, environmental, or genetic influences—may play a role.

Strength of our study are numerous. It utilizes a large and representative sample from NHANES, which enhances the generalizability of findings across different demographic groups in the U.S. population. By focusing on NHANES data, the study benefits from robust sampling techniques, which minimize selection bias and make results more applicable to the general population. Importantly, all anthropometric measurements—including weight, height, waist circumference, and body composition—were taken by trained health technicians during standardized examination visits at NHANES study centres, ensuring high measurement accuracy. We replicated the main analysis without applying survey weights and found that the results remained qualitatively unchanged, demonstrating their robustness. The study leverages accelerometer-derived sleep data, providing an objective and precise measure of sleep regularity, as opposed to relying on self-reported sleep measures. Due to its ability to capture day-to-day variations of sleep, its unbiasedness for short study periods (Fischer et al., 2021) and our large sample size, we focussed on the SRI as measure for sleep regularity. Incorporating other obesity measures enhanced our analysis, particularly across diverse racial groups, and addresses the known limitations associated with relying solely on BMI.

There are also some noteworthy limitations, key of which is the cross-sectional nature of the study, which precludes causal inference. The relationship between sleep and obesity could be bidirectional (Koolhaas et al., 2019) and we were unable to address this. Another limitation of this study is the lack of data on the specific timing and season of NHANES participants’ recruitment. Seasonal variations in light exposure and social activities (e.g., holidays in summer) may influence sleep regularity, potentially impacting the SRI. The distribution of the SRI in our sample, characterized by a non-normal, negatively skewed shape, aligns with other findings (Lunsford-Avery et al., 2018; Windred et al., 2021) although these studies reported higher SRI values overall. In our analysis, the survey-weighted median SRI was 63.89, notably lower than the median of 81 reported by Windred et al. (Windred et al., 2021). In a sample of 6,052 participants from NHANES 2011-14, Wang et al. reported higher SRIs, with a median of 84.5 (Supplementary Table 8 in (Wang et al., 2023)). The differences in SRI values may, in part, be attributed to the notably different racial composition of their sample, which included 69.2% Non-Hispanic White participants compared to 41% in our sample. In contrast, Yang et al. (Yang et al., 2024) reported a mean SRI of 63.0 among 5,589 participants from the same cohort who had slightly higher mean age (54.5 years). We report multiplication factors (i.e., percentage changes) for SRI quintiles, which are specific to our sample, limiting direct comparisons with other quantiles. To complement this analysis, we provide extrapolations across the entire SRI range. Finally, the SRI’s stability within individuals across different life phases (e.g., age or major life events) remains unknown. Without longitudinal data, it is difficult to determine if SRI is a stable measure over time or if it varies significantly within individuals as they age or undergo lifestyle changes.

In sum, our study highlights the importance of regular sleep patterns for maintaining a healthy weight. We also observed important differences in the association between regular sleep and adiposity/weight measures across gender, and ethnic groups. Future studies should confirm these subgroup findings, and investigate the stability of SRI over time within individuals, focusing on how life changes (e.g., aging, shift in employment status, or significant lifestyle alterations) affect SRI and related health outcomes like BMI. Long-term follow-up studies could help determine causality and assess the predictive power of SRI on weight gain and metabolic health over the lifespan.

## Supplementary Material

Supplementary

## Figures and Tables

**Table 1:** Characteristics of study population in NHANES 2011-14 (without survey weights) stratified by quintiles of sleep regularity index (SRI).

	Overall N = 7,085	SRI Q1 N = 1,417	SRI Q2 N = 1,417	SRI Q3 N = 1,417	SRI Q4 N = 1,417	SRI Q5 N = 1,417
**Sleep Regularity Index,** **SRI** ^1^	61 (49, 71)	36 (28, 41)	52 (49, 55)	61 (59, 64)	69 (68, 71)	78 (76, 82)
**Age**	52 (36, 65)	57 (38, 71)	53 (37, 68)	51 (35, 65)	50 (37, 64)	47 (37, 60)
**Gender**						
Male	3,333 (47%)	784 (55%)	670 (47%)	645 (46%)	639 (45%)	595 (42%)
Female	3,752 (53%)	633 (45%)	747 (53%)	772 (54%)	778 (55%)	822 (58%)
**Race/Ethnicity** ^2^						
Mexican American	826 (12%)	107 (7.6%)	149 (11%)	185 (13%)	209 (15%)	176 (12%)
Other Hispanic	682 (9.6%)	93 (6.6%)	121 (8.5%)	129 (9.1%)	179 (13%)	160 (11%)
Non-Hispanic White	2,908 (41%)	586 (41%)	560 (40%)	564 (40%)	537 (38%)	661 (47%)
Non-Hispanic Black	1,659 (23%)	464 (33%)	395 (28%)	341 (24%)	276 (19%)	183 (13%)
Non-Hispanic Asian	797 (11%)	127 (9.0%)	138 (9.7%)	155 (11%)	181 (13%)	196 (14%)
Other Race - Including Multi-Racial	213 (3.0%)	40 (2.8%)	54 (3.8%)	43 (3.0%)	35 (2.5%)	41 (2.9%)
**Education Level** ^2^						
Less Than 9th Grade	643 (9.1%)	139 (9.8%)	126 (8.9%)	121 (8.5%)	152 (11%)	105 (7.4%)
9-11th Grade (Includes 12th grade with no diploma)	972 (14%)	236 (17%)	214 (15%)	175 (12%)	178 (13%)	169 (12%)
High School Grad/GED or Equivalent	1,566 (22%)	331 (23%)	321 (23%)	345 (24%)	289 (20%)	280 (20%)
Some College or AA degree	2,125 (30%)	465 (33%)	460 (32%)	415 (29%)	414 (29%)	371 (26%)
College Graduate or above	1,779 (25%)	246 (17%)	296 (21%)	361 (25%)	384 (27%)	492 (35%)
**Household Income** ^2^						
< $20,000	1,629 (23%)	483 (34%)	369 (26%)	322 (23%)	254 (18%)	201 (14%)
≥ $20,000	5,456 (77%)	934 (66%)	1,048 (74%)	1,095 (77%)	1,163 (82%)	1,216 (86%)
**Occupational Category** ^2^						
Working	3,646 (51%)	453 (32%)	612 (43%)	760 (54%)	834 (59%)	987 (70%)
Retired or Student	1,683 (24%)	470 (33%)	399 (28%)	317 (22%)	288 (20%)	209 (15%)
Unable to Work (Health or Family Reasons)	1,262 (18%)	368 (26%)	282 (20%)	239 (17%)	218 (15%)	155 (11%)

Unemployed	326 (4.6%)	81 (5.7%)	79 (5.6%)	67 (4.7%)	52 (3.7%)	47 (3.3%)
Other	168 (2.4%)	45 (3.2%)	45 (3.2%)	34 (2.4%)	25 (1.8%)	19 (1.3%)
**Marital Status** ^2^						
Married	3,608 (51%)	528 (37%)	671 (47%)	720 (51%)	783 (55%)	906 (64%)
Widowed	642 (9.1%)	191 (13%)	144 (10%)	121 (8.5%)	107 (7.6%)	79 (5.6%)
Divorced	824 (12%)	203 (14%)	178 (13%)	160 (11%)	163 (12%)	120 (8.5%)
Separated	235 (3.3%)	68 (4.8%)	44 (3.1%)	38 (2.7%)	51 (3.6%)	34 (2.4%)
Never married	1,297 (18%)	340 (24%)	292 (21%)	264 (19%)	221 (16%)	180 (13%)
Living with partner	479 (6.8%)	87 (6.1%)	88 (6.2%)	114 (8.0%)	92 (6.5%)	98 (6.9%)
**Alcohol Consumption** ^2^						
Heavy Drinker	176 (2.5%)	47 (3.3%)	39 (2.8%)	37 (2.6%)	31 (2.2%)	22 (1.6%)
Moderate Drinker	5,685 (80%)	1,113 (79%)	1,134 (80%)	1,156 (82%)	1,116 (79%)	1,166 (82%)
Never/Non-Drinker	1,224 (17%)	257 (18%)	244 (17%)	224 (16%)	270 (19%)	229 (16%)
**Smoking Status** ^2^						
Heavy smoker	769 (11%)	230 (16%)	174 (12%)	148 (10%)	121 (8.5%)	96 (6.8%)
Light smoker	452 (6.4%)	145 (10%)	105 (7.4%)	85 (6.0%)	67 (4.7%)	50 (3.5%)
Moderate smoker	137 (1.9%)	32 (2.3%)	40 (2.8%)	18 (1.3%)	28 (2.0%)	19 (1.3%)
Non smokers	3,976 (56%)	666 (47%)	734 (52%)	792 (56%)	865 (61%)	919 (65%)
Previous smoker	1,751 (25%)	344 (24%)	364 (26%)	374 (26%)	336 (24%)	333 (24%)
**Vitamin D Level** ^1^	63 (46, 82)	58 (40, 79)	62 (43, 81)	63 (46, 83)	64 (48, 81)	68 (53, 85)
**Total Caloric Intake** ^1^	1,917 (1,441, 2,545)	1,824 (1,341, 2,497)	1,861 (1,370, 2,457)	1,936 (1,448, 2,575)	1,942 (1,472, 2,556)	1,998 (1,548, 2,604)
**Depression Score (PHQ-9)** ^1^	2.0 (0.0, 4.0)	3.0 (0.0, 7.0)	2.0 (0.0, 5.0)	2.0 (0.0, 4.0)	1.0 (0.0, 3.0)	1.0 (0.0, 3.0)
**Activity Level** ^1^	7.7 (5.1, 10.8)	5.1 (3.1, 7.6)	6.8 (4.5, 9.4)	8.0 (5.5, 10.7)	9.1 (6.5, 12.0)	9.9 (7.2, 13.1)

1 Median (Q1, Q3)

2 n (%)

**Table 2:** Overview of obesity measures in the study population (without survey weights) stratified by quintiles of the Sleep Regularity Index (SRI). Total Fat Mass, Fat Mass Index and Percent Body Fat (%) have high missingness (48%).

	Median (IQR)^1^	SRI Q1 N = 1,417^1^	SRI Q2 N = 1,417^1^	SRI Q3 N = 1,417^1^	SRI Q4 N = 1,417^1^	SRI Q5 N = 1,417^1^	Missingness (%)
**Body Mass Index (BMI)**	28 (24, 33)	29 (25, 35)	28 (25, 33)	28 (25, 33)	28 (24, 32)	27 (23, 31)	1.04%
**Waist Circumference (cm)**	98 (88, 109)	102 (91, 114)	99 (89, 111)	99 (89, 110)	97 (87, 108)	94 (85, 104)	4.11%
**A Body Shape Index (ABSI)**	0.82 (0.79, 0.85)	0.83 (0.79, 0.86)	0.82 (0.79, 0.86)	0.82 (0.79, 0.85)	0.82 (0.79, 0.85)	0.81 (0.79, 0.84)	4.43%
**Waist-to-Height Ratio (WHtR)**	0.59 (0.53, 0.66)	0.61 (0.54, 0.68)	0.60 (0.54, 0.66)	0.60 (0.53, 0.66)	0.58 (0.52, 0.65)	0.56 (0.52, 0.62)	4.30%
**Sagittal Abdominal Diameter (cm)**	22.5 (19.6, 25.7)	23.9 (20.7, 27.2)	22.9 (20.1, 26.0)	22.9 (19.8, 26.0)	22.2 (19.5, 25.1)	21.2 (18.6, 24.1)	7.18%
**SAD-to-Height Ratio (SADHtR)**	0.135 (0.118, 0.154)	0.143 (0.123, 0.162)	0.137 (0.120, 0.156)	0.137 (0.119, 0.155)	0.133 (0.117, 0.152)	0.127 (0.112, 0.145)	7.28%
**Visceral Adiposity Index (VAI)**	1.74 (1.01, 3.03)	1.91 (1.09, 3.32)	1.80 (1.07, 3.09)	1.77 (1.00, 3.09)	1.66 (0.95, 3.06)	1.57 (0.93, 2.63)	8.93%
**Total Fat Mass (kg)**	26 (20, 34)	27 (19, 36)	26 (19, 34)	27 (20, 36)	26 (20, 33)	24 (19, 31)	48.64%
**Fat Mass Index (FMI)**	9.2 (6.8, 12.4)	9.4 (6.6, 13.2)	9.2 (6.7, 12.5)	9.9 (7.2, 13.2)	9.3 (6.8, 12.4)	8.6 (6.8, 11.1)	48.71%
**Percent Body Fat (%)**	33 (27, 40)	32 (26, 40)	33 (27, 41)	35 (28, 41)	34 (27, 40)	33 (27, 39)	48.64%
**Body Roundness Index (BRI)**	5.18 (3.91, 6.76)	5.74 (4.18, 7.36)	5.35 (4.12, 6.95)	5.32 (3.98, 6.82)	5.05 (3.84, 6.60)	4.61 (3.66, 5.96)	4.30%
**Lipid Accumulation Product (LAP)**	51 (27, 91)	59 (32, 105)	53 (30, 95)	52 (28, 95)	48 (26, 87)	44 (24, 75)	8.64%

1 Median (Q1, Q3)

**Table 3:** Multiplicative effects (i.e., percentage changes) and 95% bootstrap confidence intervals of SRI quintile-changes on BMI.

Model	Race/Ethnicity	Sex	Q1 → Q2	Q1 → Q3	Q1 → Q4	Q1 → Q5
Model 1			0.969 (0.963; 0.976)	0.952 (0.942; 0.963)	0.938 (0.925; 0.951)	0.9225 (0.906; 0.939)
Model 2			0.981 (0.973; 0.988)	0.97 (0.958; 0.982)	0.96 (0.945; 0.976)	0.9503 (0.932; 0.97)
Model 3		Male	0.993 (0.985; 1.001)	0.988 (0.976; 1.001)	0.985 (0.969; 1.002)	0.981 (0.961; 1.002)
		Female	0.969 (0.959; 0.979)	0.951 (0.937; 0.967)	0.936 (0.918; 0.957)	0.92 (0.898; 0.945)
Model 4	Mexican American	Male	1.015 (1; 1.03)	1.024 (1; 1.048)	1.032 (1; 1.063)	1.04 (1; 1.081)
		Female	0.992 (0.973; 1.013)	0.987 (0.958; 1.021)	0.984 (0.945; 1.027)	0.98 (0.931; 1.034)
	Other Hispanic	Male	0.997 (0.978; 1.015)	0.995 (0.965; 1.023)	0.993 (0.955; 1.031)	0.991 (0.943; 1.039)
		Female	0.974 (0.95; 0.994)	0.959 (0.923; 0.991)	0.947 (0.9; 0.989)	0.934 (0.875; 0.986)
	Non-Hispanic White	Male	0.987 (0.978; 0.997)	0.98 (0.965; 0.995)	0.973 (0.955; 0.993)	0.967 (0.943; 0.991)
		Female	0.964 (0.953; 0.974)	0.945 (0.927; 0.96)	0.928 (0.905; 0.948)	0.91 (0.882; 0.934)
	Non-Hispanic Black	Male	1.014 (1; 1.027)	1.021 (0.999; 1.042)	1.028 (0.999; 1.056)	1.036 (0.999; 1.071)
		Female	0.99 (0.976; 1.004)	0.985 (0.963; 1.006)	0.98 (0.952; 1.007)	0.975 (0.94; 1.009)
	Non-Hispanic Asian	Male	0.992 (0.974; 1.007)	0.987 (0.96; 1.011)	0.984 (0.948; 1.015)	0.979 (0.935; 1.019)
		Female	0.969 (0.954; 0.987)	0.952 (0.929; 0.98)	0.938 (0.908; 0.973)	0.922 (0.886; 0.967)
	Other Race - Including Multi-Racial	Male	0.975 (0.942; 1.013)	0.961 (0.91; 1.02)	0.949 (0.884; 1.026)	0.937 (0.856; 1.033)
		Female	0.952 (0.917; 0.994)	0.927 (0.872; 0.991)	0.905 (0.836; 0.988)	0.882 (0.798; 0.984)

Model 1 was adjusted for age and sex; model 2 additionally for race/ethnicity, education level, household income, occupational category, marital status, alcohol consumption, smoking status, vitamin D level, total caloric intake, PHQ-9 depression score and activity level); model 3 further includes interaction terms sex and model 4 further included interaction terms for race/ethnicity (for additional information see also Suppl. Fig. 5S&6S).

Parentheses (x; y) represent 95% bootstrap confidence intervals.

## Data Availability

All non-processed data are freely available to download at https://www.cdc.gov/Nchs/Nhanes/. R-Codes are available on request from the corresponding author.
