# Supplementary material for: Sex- and ethnic differences in the cross-sectional association between sleep regularity and obesity among US adults, NHANES 2011-2014
Source: J Biol Rhythms. Author manuscript; Available in PMC 2026 Jan 17. (PMC7618617; doi:10.1177/07487304251391267)
Supplement: Supplementary [file EMS209612-supplement-Supplementary.pdf]

**Supplementary Material:**

# Racial/ethnic and sex differences in the association between Sleep Regularity Index (SRI) and obesity measures in adults: NHANES 2011-2014

**Jürgen Degenfellner [1,2*], Susanne Strohmaier [1], Eva Schernhammer [1, 4, 5]**

1. Department of Epidemiology, Center of Public Health, Medical University of Vienna, Vienna, Austria,
2. Institute of Physiotherapy, ZHAW School of Health Sciences, Katharina-Sulzer-Platz 9, Winterthur, Switzerland
4. Channing Division of Network Medicine, Brigham and Women's Hospital and Harvard Medical School, Boston, USA
5. Department of Epidemiology, Harvard T.H. Chan School of Public Health, Boston, USA

* Correspondence: degn@zhaw.ch

**Detailed information on covariables used in regression models:**

*Race/ethnicity* in the NHANES 2011-2014 cycles included the categories 'Mexican American'/'Other Hispanic'/'Non-Hispanic White'/Non-Hispanic Black'/'Non-Hispanic Asian'/'Other Race - Including Multi-Racial'. Sex/*Gender* was included as biological sex with levels Female/Male.

The *occupational* category was defined using OCD150 (Type of Work Done Last Week) and OCQ380 (Main Reason Did Not Work Last Week) with the following categories: 'Working', which includes participants working at a job or business or with a job but not at work; 'retired or student', which combines participants who are retired or going to school; 'unable to work (health or family reasons)'; 'unemployed', which includes participants looking for work or on layoff; and 'other', which includes participants with unspecified reasons.

*Education* represents the highest educational attainment for adults aged 20 and older. Responses 'Refused' and 'Don't Know' were set to missing values. The remaining responses were categorized as 'Less Than 9th Grade'/'9-11th Grade (including 12th grade with no diploma)'/'High School Graduate/GED or Equivalent'/'Some College or Associate's Degree and 'College Graduate or above'.

*Annual Household Income* captures the household income range. Responses 'Refused' and 'Don't Know' were set to missing values. The remaining responses were categorized into income ranges as follows: 'Less 20,000'/'Greater or equal $20,000'.

*Smoking status* for participants who reported having smoked less than 100 cigarettes in their lifetime were classified as 'Non-smokers.' Among those who reported having smoked at least 100 cigarettes, further classification was done based on current smoking behaviour. Participants who indicated they do not currently smoke cigarettes were classified as 'Previous smokers.' For current smokers, smoking intensity was assessed using the average number of cigarettes smoked per day over the past 30 days. Based on this, current smokers were categorized as 'Light smokers' (1-5 cigarettes/day), 'Moderate smokers' (6-9 cigarettes/day), or 'Heavy smokers' (10 or more cigarettes/day). 'Refused' or 'Don't Know' responses were coded as missing values.

*Alcohol consumption* was categorized into Never/Non-Drinker, Moderate Drinker, and Heavy Drinker, based on responses to the NHANES alcohol use questions. Participants who reported

never consuming at least 12 alcoholic drinks in their lifetime were classified as Never/Non-Drinkers. Heavy Drinkers were defined as individuals exceeding sex-specific thresholds for alcohol consumption: 8 drinks or more per week (or equivalent monthly or yearly consumption) for females and 15 drinks or more per week (or equivalent) for males. Moderate Drinkers were defined as those consuming alcohol below the heavy drinking thresholds, including up to 1 drink per day for females and up to 2 drinks per day for males. Responses indicating “Refused” or “Don’t Know” were treated as missing values.

*Marital status* was determined based on participant responses, with any ‘Refused’ or ‘Don’t Know’ entries set to missing values. The remaining responses were classified into six categories: Married/ Widowed/Divorced/Separated/Never married/Living with partner.

*Depression* was assessed using the PHQ-9 score (Kroenke & Spitzer, 2002), ranging from 0 to 27 with higher scores indicating more severe depressive symptoms. Responses ‘Refused’ or ‘Don’t Know’ in the score-defining items were set to missing.

*Activity level* was quantified using the participant’s MIMS triaxial value per minute. Only positive values were retained to focus on active periods. For each participant, we calculated the median as a robust measure less influenced by outliers and skewness.

*Vitamin D levels* were included as 25-hydroxyvitamin D2 and D3 in nmol/L which was measured using liquid chromatography-tandem mass spectrometry (Herrick et al., 2019).

*Total caloric intake* was assessed using data from the NHANES total nutrient intakes file, which captures dietary intake information based on a 24-hour dietary recall interview. This included all foods and beverages (including water) consumed from midnight to midnight during the day prior to the interview. The reported total caloric intake (in kcal) reflects the estimated energy intake for that period. To address potential outliers, caloric intake values were winsorized at the 1st and 99th percentiles. Missing values were retained as such.

In addition, we derived two variables related to *meal timing variability* between weekdays (Monday to Thursday) and weekends (Friday to Sunday). Specifically, we calculated the difference in the timing of the last meal and the difference in the eating time window (i.e., the duration between the first and last meal) between these periods. Both variables were winsorized at the 1st and 99th percentiles to limit the influence of outliers. Due to approximately 38% missingness, these variables were only included in a sensitivity analysis and not in the main regression models.

Covariables were imputed using k-Nearest-Neighbours before estimating the regression models (Kowarik & Templ, 2016).

**Figure 1S**: Flowchart illustrating the filtering steps applied separately to the NHANES 2011–2012 and NHANES 2013–2014 public release accelerometer datasets. The filtering process included several steps: exclusion of participants younger than 20 years, removal of individuals without an available Physical Activity Monitor (PAM) file, and application of multiple data quality filters—including a 60-second epoch filter, non-wear time filter, time step consistency filter, consecutive days requirement, and missingness rate threshold (PAXPREDM = NA/non-wear/unknown). After applying these filters, the final analytical sample sizes were 3,677 participants for NHANES 2011–2012 and 3,408 participants for NHANES 2013–2014.

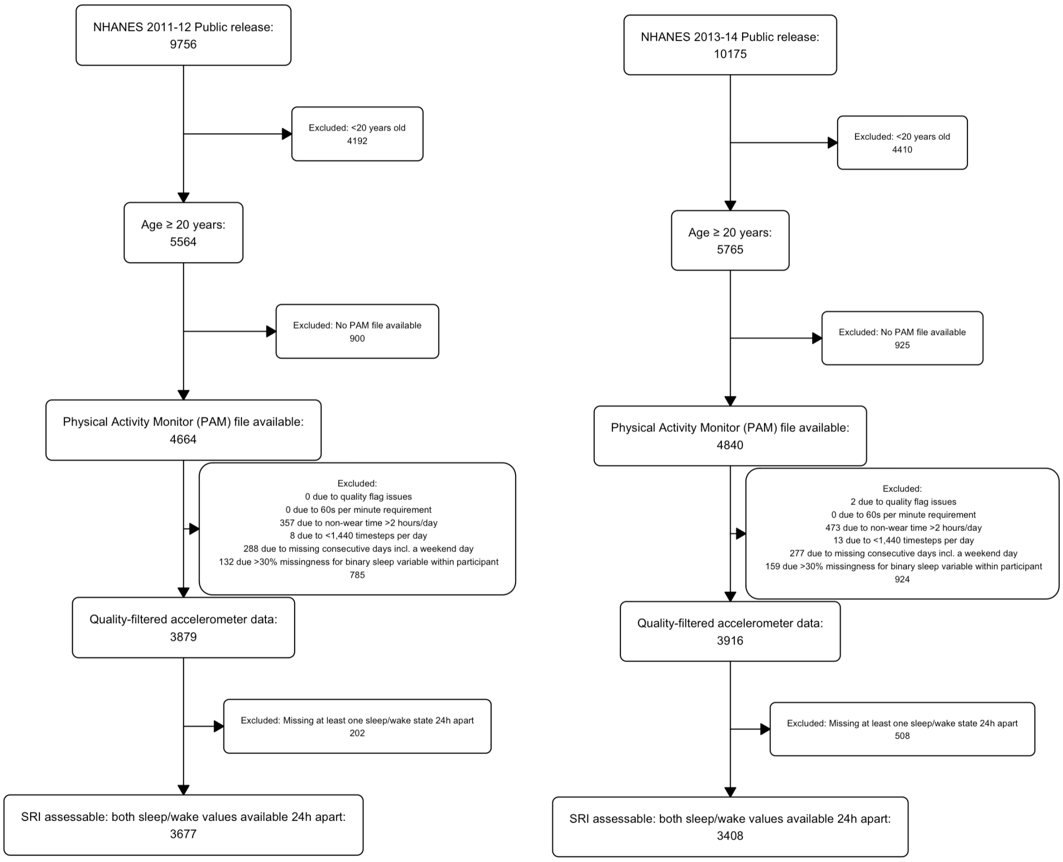

**Figure 2S**. Exemplary visualizations of the binary sleep variable (1=sleep, 0=wake) relating to different SRI values in NHANES 2013-14. Red indicates the 24h marks, green, and turquoise were classified as 'non-wear' and 'unknown' respectively by open-source machine learning algorithm (CDC; CDC). **Top panel**: Sleep-wake pattern for SRI around 0 showing very irregular sleep. **Middle panel**: SRI around 45 is related to a more regular sleep pattern. **Bottom panel**: Very high SRI value of approximately 92 for highly regular sleep. Note, that also majorily 'sleep' or only 'wake' states would result in high SRI values.

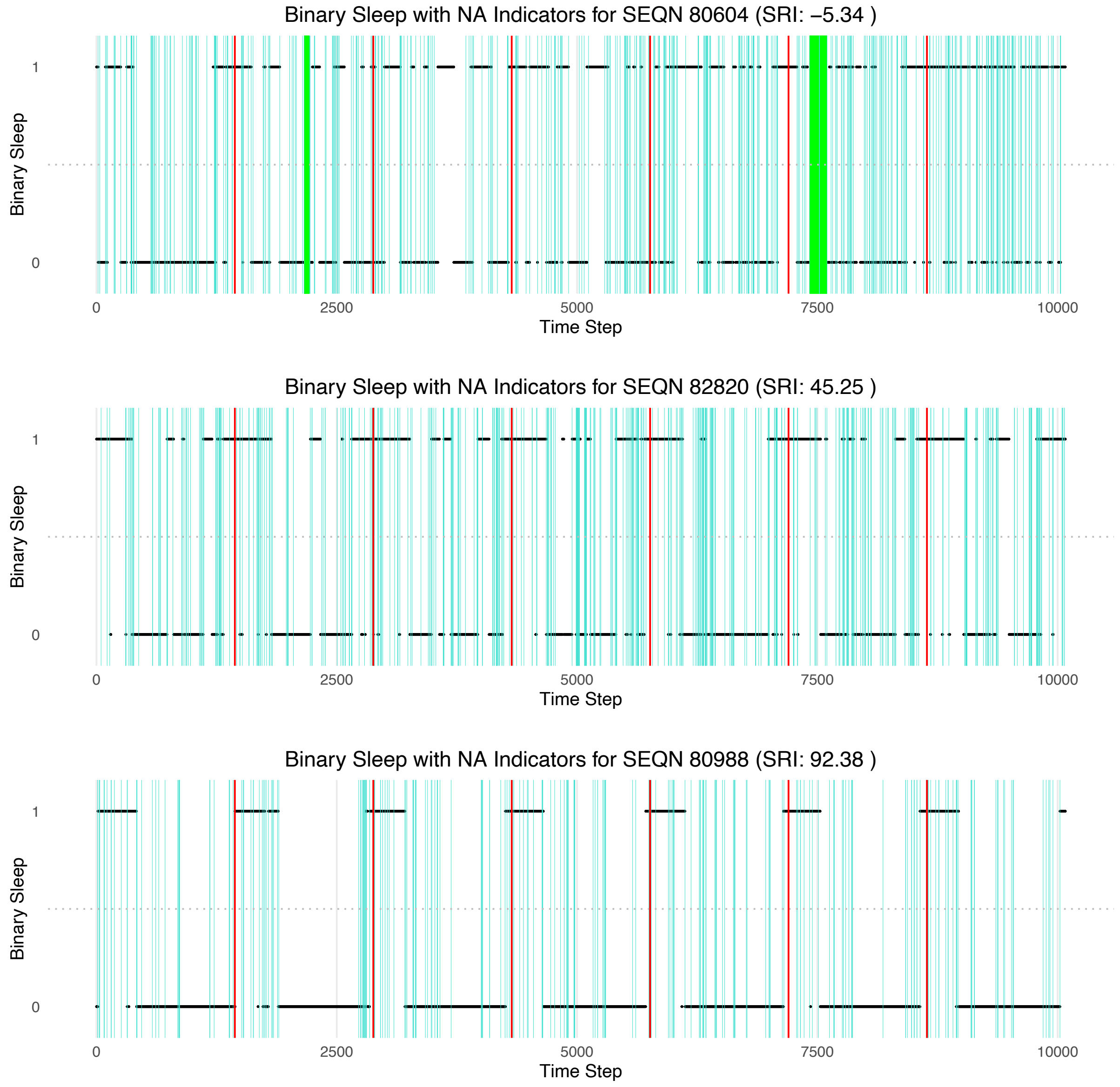

**Figure 3S**. Comparison of original and winsorized BMI. To limit the influence of extreme values, we applied winsorization to the BMI. Values below the 1st percentile (18.01 kg/m$^2$) and above the 99th percentile (51.80 kg/m$^2$) were capped at these respective thresholds and set to the respective nearest quantiles.

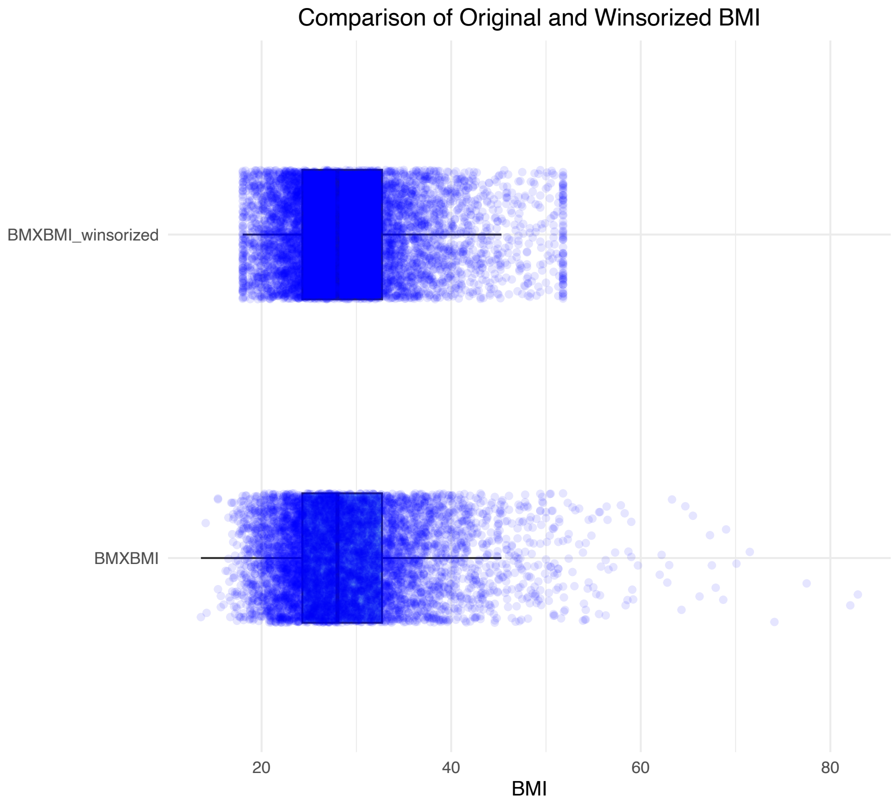

**Figure 4S.** Hypothesized relationships between sleep regularity index (SRI, exposure) and body mass index (BMI, outcome), with various sociodemographic, lifestyle, and health-related covariables. Variables are grouped by conceptual domains and color-coded: non-modifiable factors (age, sex, ethnicity), modifiable lifestyle factors (smoking, alcohol consumption, physical activity, caloric intake, vitamin D), socioeconomic status (SES) indicators (education, occupation, household income, marital status), and depression. The spatial layout of circles does not represent statistical distances or clustering but is chosen for clarity of presentation. Each arrow depicts a hypothesized direct relationship informed by prior literature and theory. Thus, the figure is intended as a conceptual framework for variable inclusion and adjustment in the analysis, not as a graphical display of empirical results.

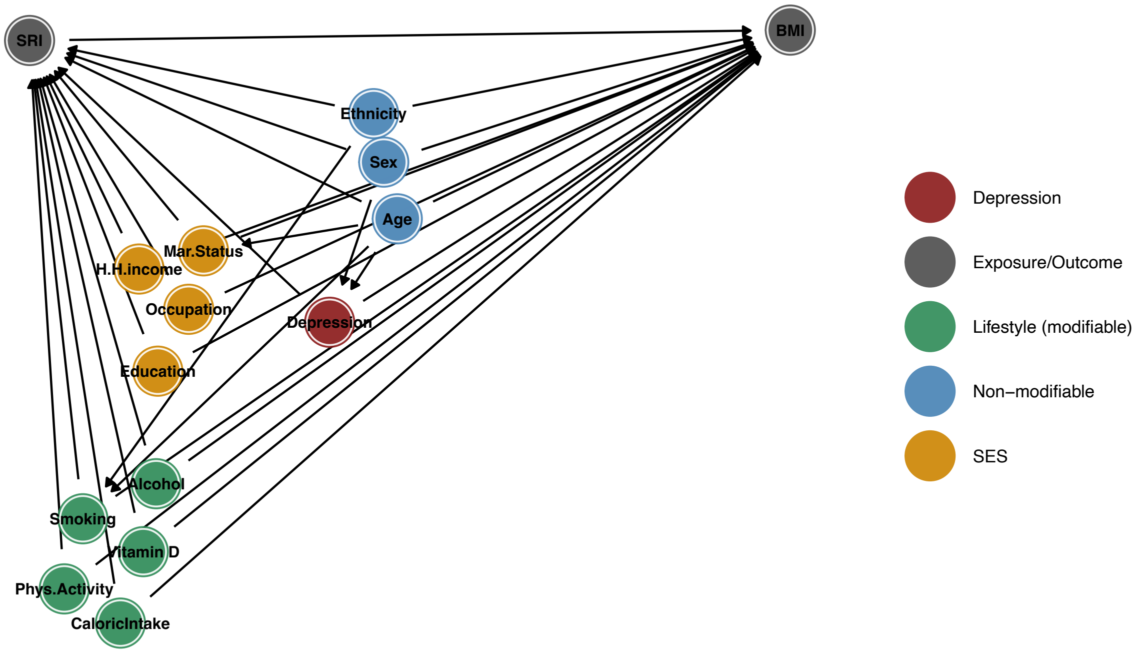

**Figure 5S.** Model equation for fully adjusted model of log(BMI) (without survey weights).

$$log(\text{BMI}_i) = \beta_0 + \beta_1\text{SRI}_i + \beta_2\text{Ethnicity}_i + \beta_3(\text{SRI} \times \text{Ethnicity})_i$$

$$+\beta_4\text{Sex}_i + \beta_5(\text{SRI} \times \text{Sex})_i$$

$$+\beta_6\text{Education}_i + \beta_7\text{Household Income}_i + \beta_8\text{Alcohol Consumption}_i$$

$$+\beta_9\text{Smoking status}_i + \beta_{10}\text{Vitamin D}_i + \beta_{11}\text{Total caloric intake} + \beta_{12}\text{Depression score}_i$$

$$+\beta_{13}\text{Marital status}_i + \beta_{14}\text{Activity level}_i + \varepsilon_i$$

$$i = 1, \dots, n$$

$$\varepsilon_\text{i} \sim \text{N}(0, \sigma^2)$$

**Interpretation of regression coefficients in a model with log-outcome:**

Without loss of generality let $\mathbb{E}(log(Y)) = \beta_0 + \beta_1 x_1 + \cdots + \beta_k x_k$

$$\implies \beta_0 + \beta_1 x_1 + \cdots + \beta_k(x_k + 1) = \beta_0 + \beta_1 x_1 + \cdots + \beta_k x_k + \beta_k =$$

$$\beta_0 + \beta_1 x_1 + \cdots + \beta_k x_k + \beta_k = log(Y) + \beta_k = log(Y) + log(e^{\beta_k}) = log(Ye^{\beta_k})$$

Hence, a change of 1 unit in $x_k$ is (on average) a change from $Y$ to $Ye^{\beta_k}$.

**Figure 6S.** Regression modelling assumptions ((Westfall & Arias, 2020), p. 93) for model 4 (estimating the association of sleep regularity index (SRI) and BMI including interaction terms for SRI with sex and SRI with ethnicity). **Upper left**: Linearity assumption is fulfilled. **Upper right**: Some heteroskedasticity is present. **Lower left**: Independence assumption is fulfilled. **Lower right**: Normality of residuals might be slightly violated.

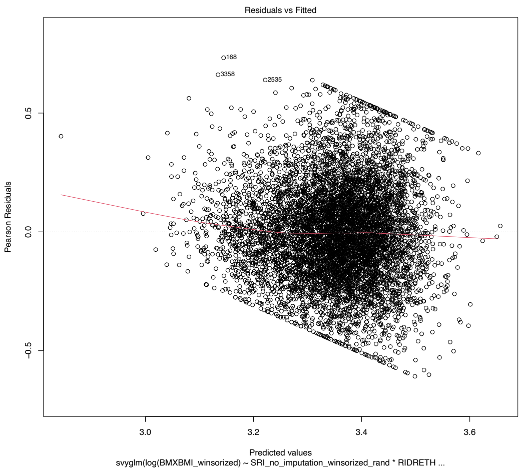


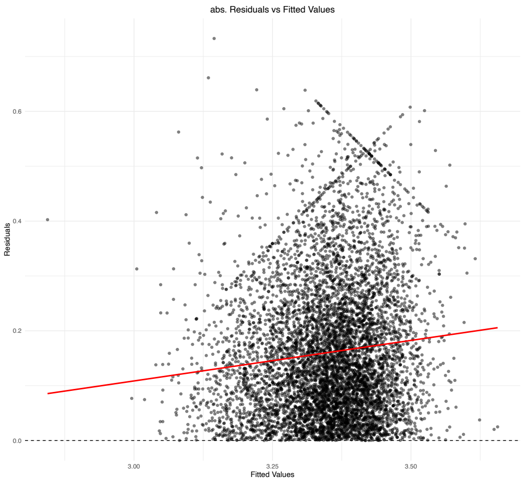


```
> cor.test(resid, lag.resid) # ns

        Pearson's product-moment correlation

data:  resid and lag.resid
t = 0.53345, df = 7082, p-value = 0.5937
alternative hypothesis: true correlation is not equal to 0
95 percent confidence interval:
 -0.01695122  0.02962185
sample estimates:
        cor
0.006338752
```

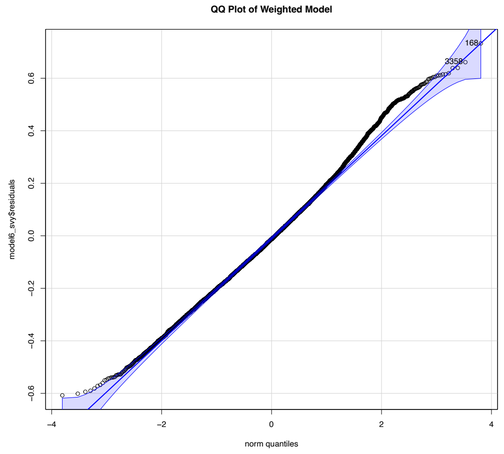

**Figure 7S.** Missingness across the analytic data set using (Tierney, 2017). Proportions of missing data (main outcome and covariables) range from 0% to 24%, with the highest missingness observed for alcohol consumption (24%). BMI has 10% missing data. Missingness for additional obesity measures can be found in table 2.

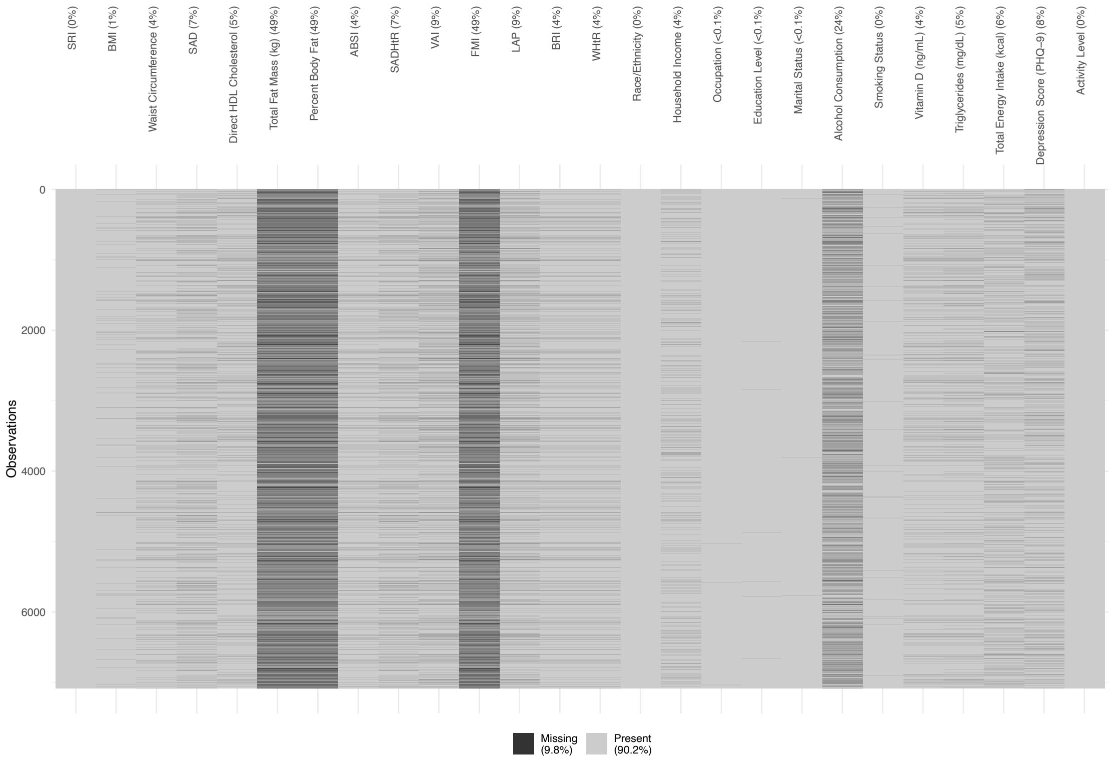

**Figure 8S.** Model predictions for BMI from model 4 (estimating the association of sleep regularity index (SRI) and BMI including interaction terms for SRI with sex and SRI with ethnicity) for 200,000 randomly selected covariable values with 95% quantiles within 5 point ranges of SRI, stratified by sex/gender and race/ethnicity. Loess smothing was used for trend lines.

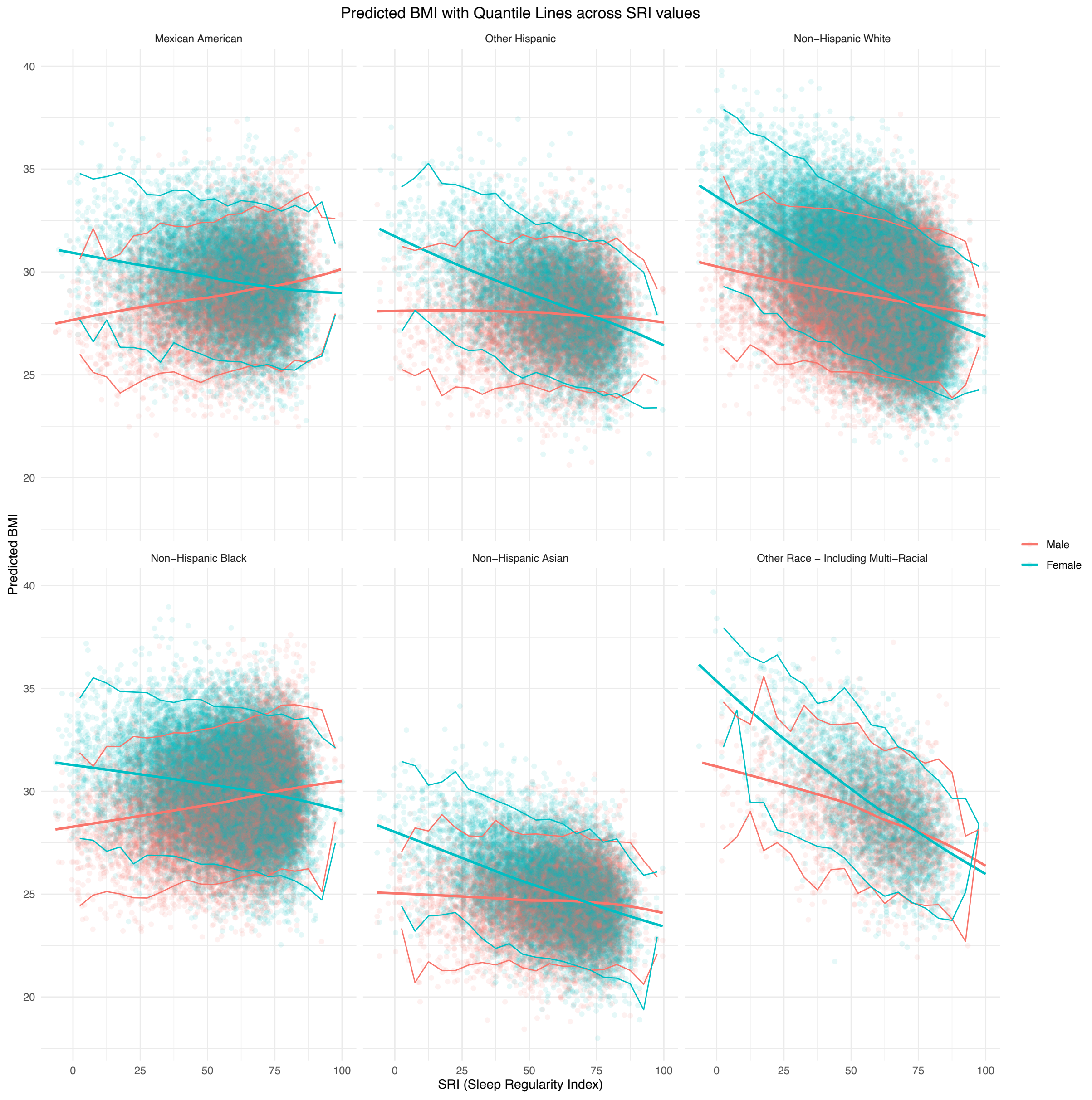

**Figure 9S.** Correlation plots for obesity outcomes stratified by sex/gender.

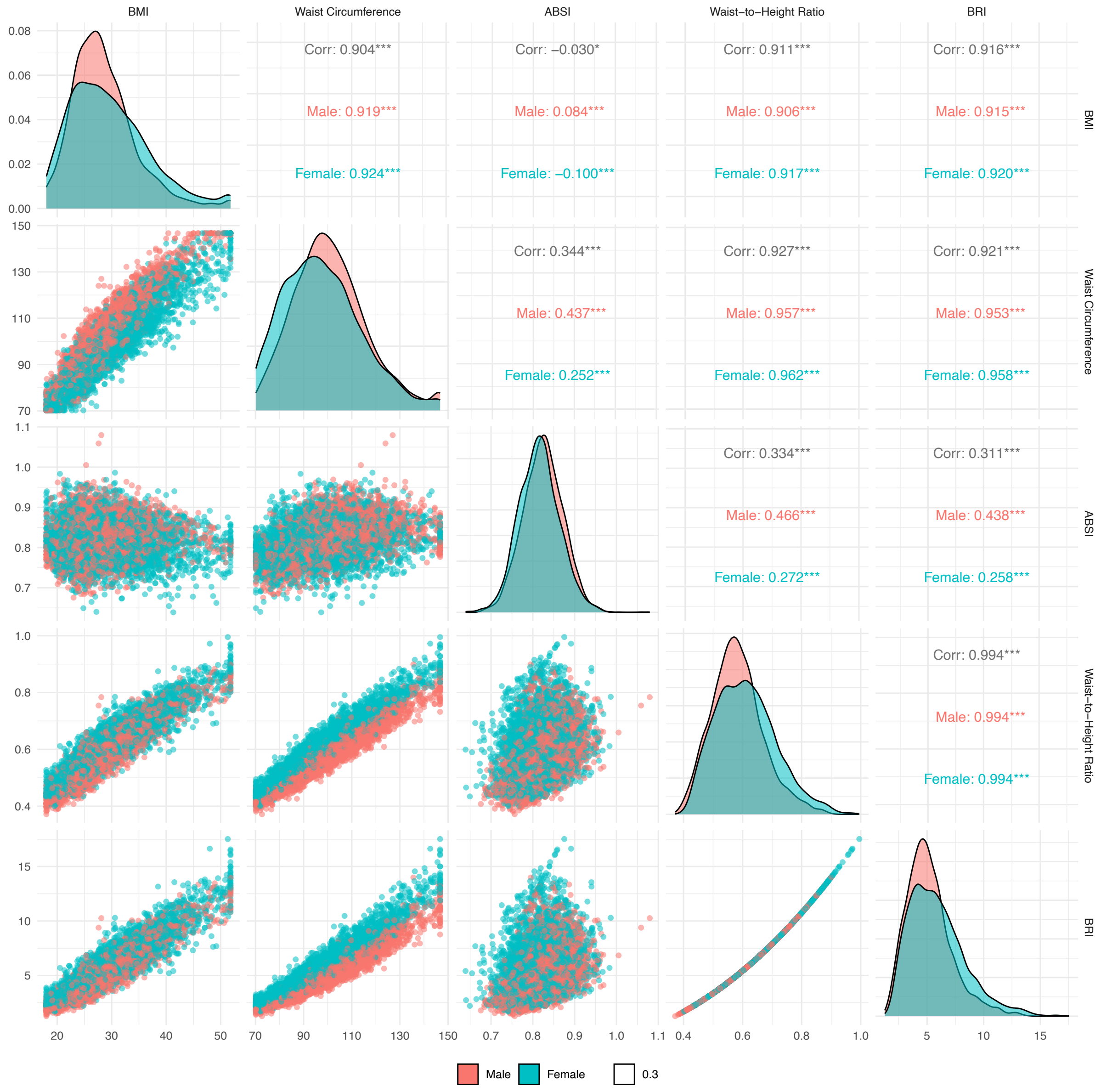

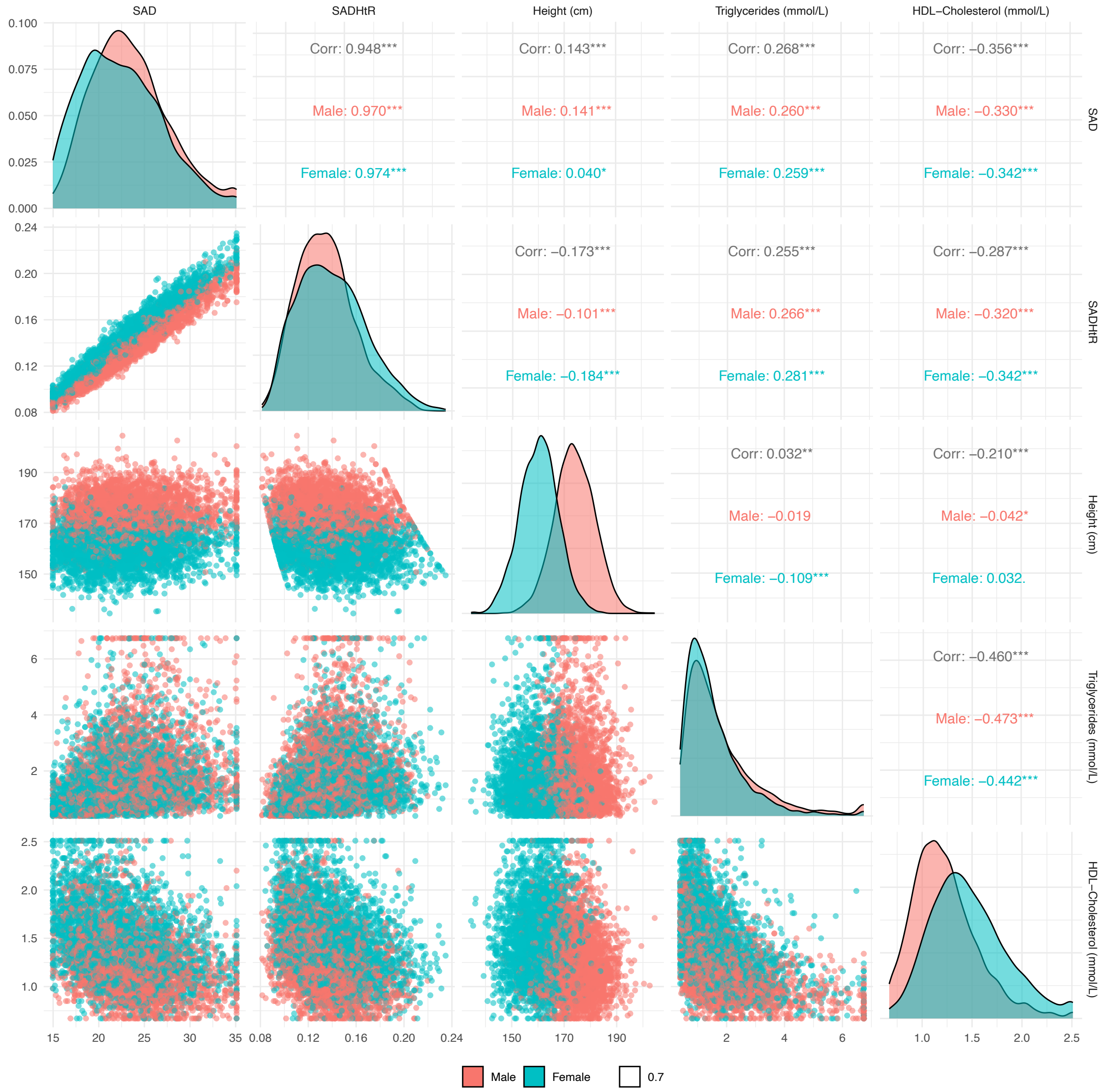
SAD
SADHtR
Height (cm)
Triglycerides (mmol/L)
HDL−Cholesterol (mmol/L)
Corr: 0.948***
Male: 0.970***
Female: 0.974***
Corr: 0.143***
Male: 0.141***
Female: 0.040*
Corr: 0.268***
Male: 0.260***
Female: 0.259***
Corr: −0.356***
Male: −0.330***
Female: −0.342***
Corr: −0.173***
Male: −0.101***
Female: −0.184***
Corr: 0.255***
Male: 0.266***
Female: 0.281***
Corr: −0.287***
Male: −0.320***
Female: −0.342***
Corr: 0.032**
Male: −0.019
Female: −0.109***
Corr: −0.210***
Male: −0.042*
Female: 0.032.
Corr: −0.460***
Male: −0.473***
Female: −0.442***
Male
Female
0.7

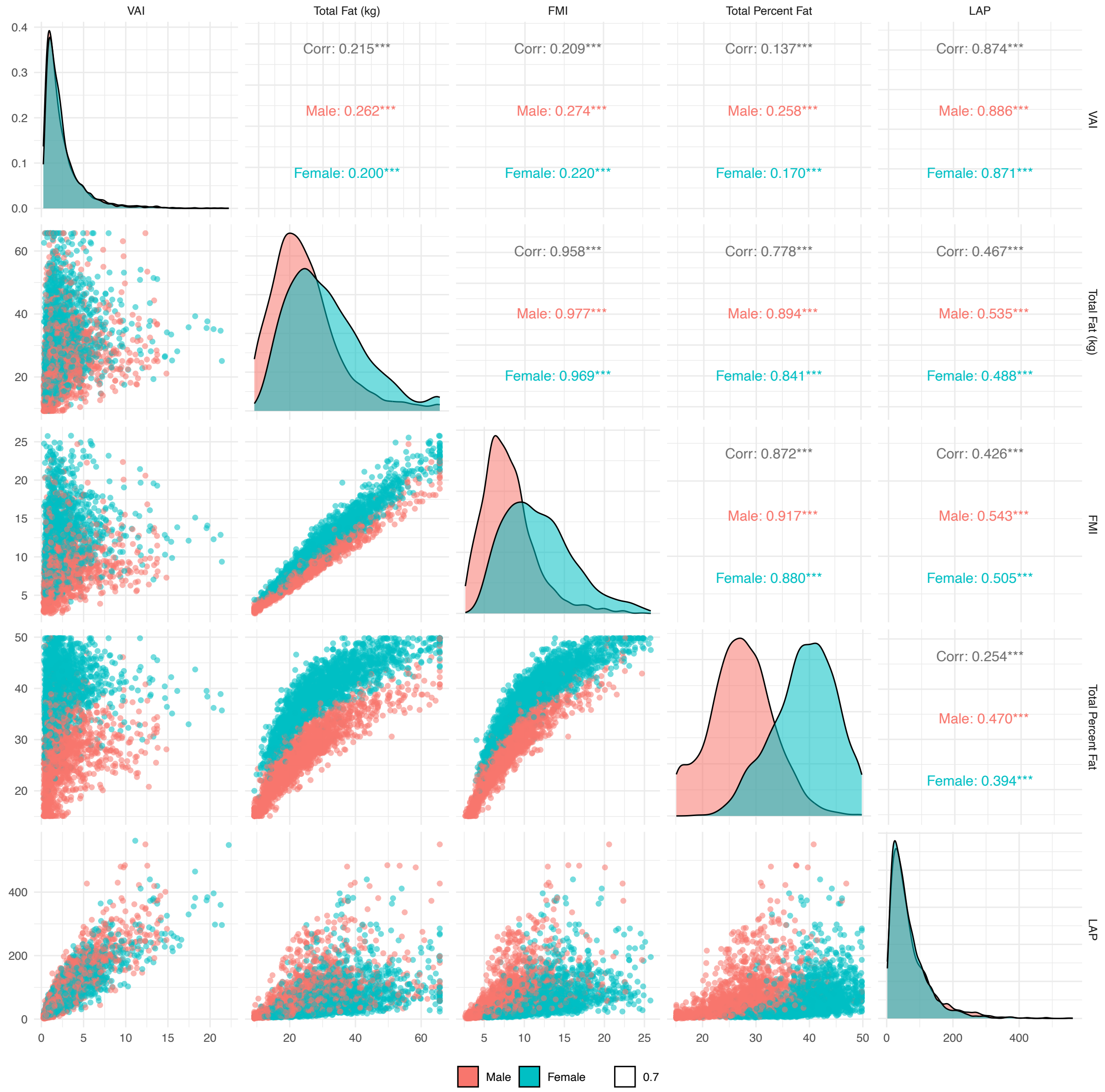

VAI
Total Fat (kg)
FMI
Total Percent Fat
LAP
Corr: 0.215***
Male: 0.262***
Female: 0.200***
Corr: 0.209***
Male: 0.274***
Female: 0.220***
Corr: 0.137***
Male: 0.258***
Female: 0.170***
Corr: 0.874***
Male: 0.886***
Female: 0.871***
Corr: 0.958***
Male: 0.977***
Female: 0.969***
Corr: 0.778***
Male: 0.894***
Female: 0.841***
Corr: 0.467***
Male: 0.535***
Female: 0.488***
Corr: 0.872***
Male: 0.917***
Female: 0.880***
Corr: 0.426***
Male: 0.543***
Female: 0.505***
Corr: 0.254***
Male: 0.470***
Female: 0.394***
Male
Female
0.7

**Figure 10S.** Fully adjusted model predictions from model 4 for other obesity measures (see table 2) with 95% (bootstrap) confidence bands stratified by sex/gender and race/ethnicity. Total fat mass (r=0.9), WHtR (r=0.9), BRI (r=0.92) and WC (r=0.9) were highly correlated with BMI. ABSI was uncorrelated with BMI (r=-0.03).

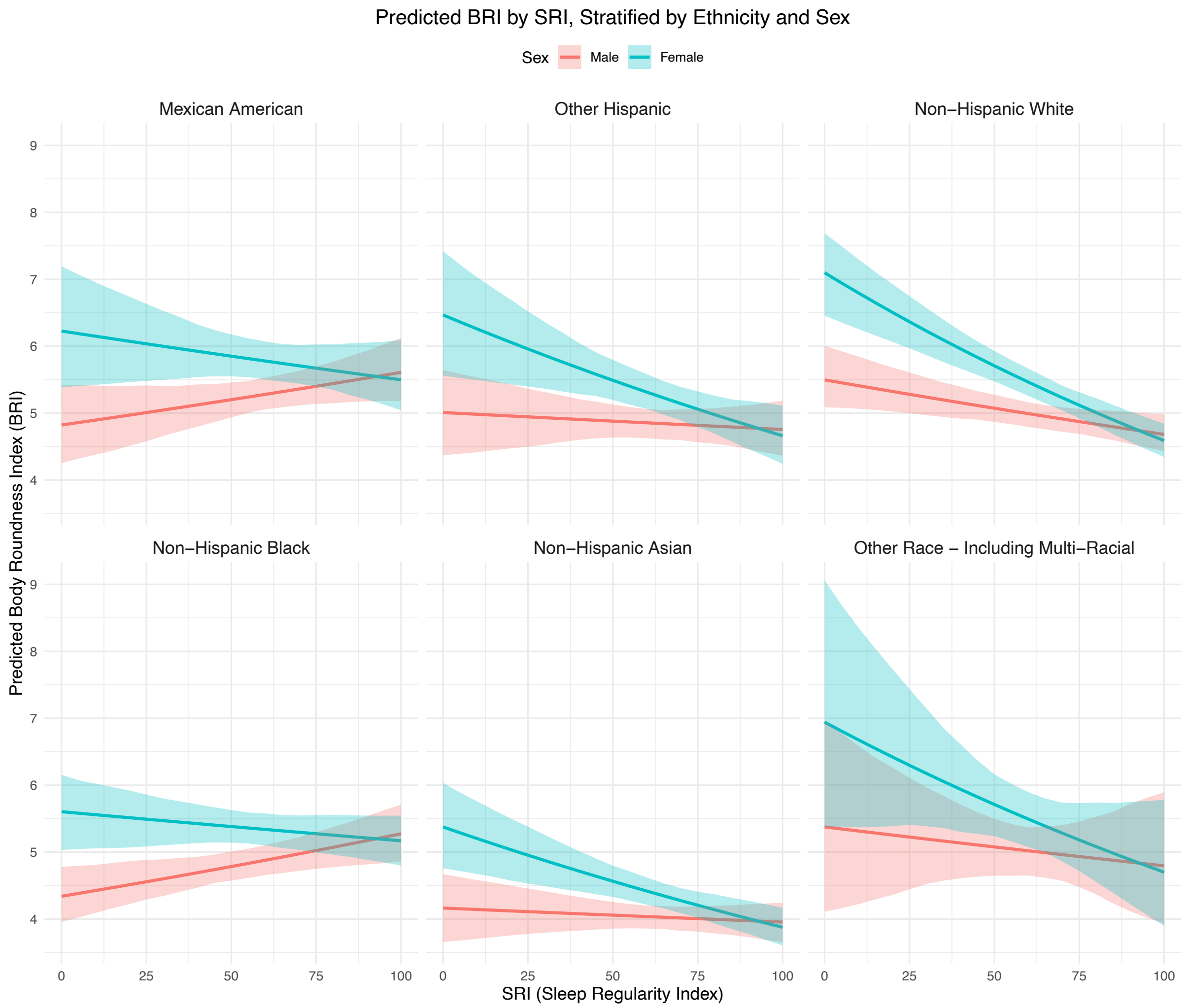

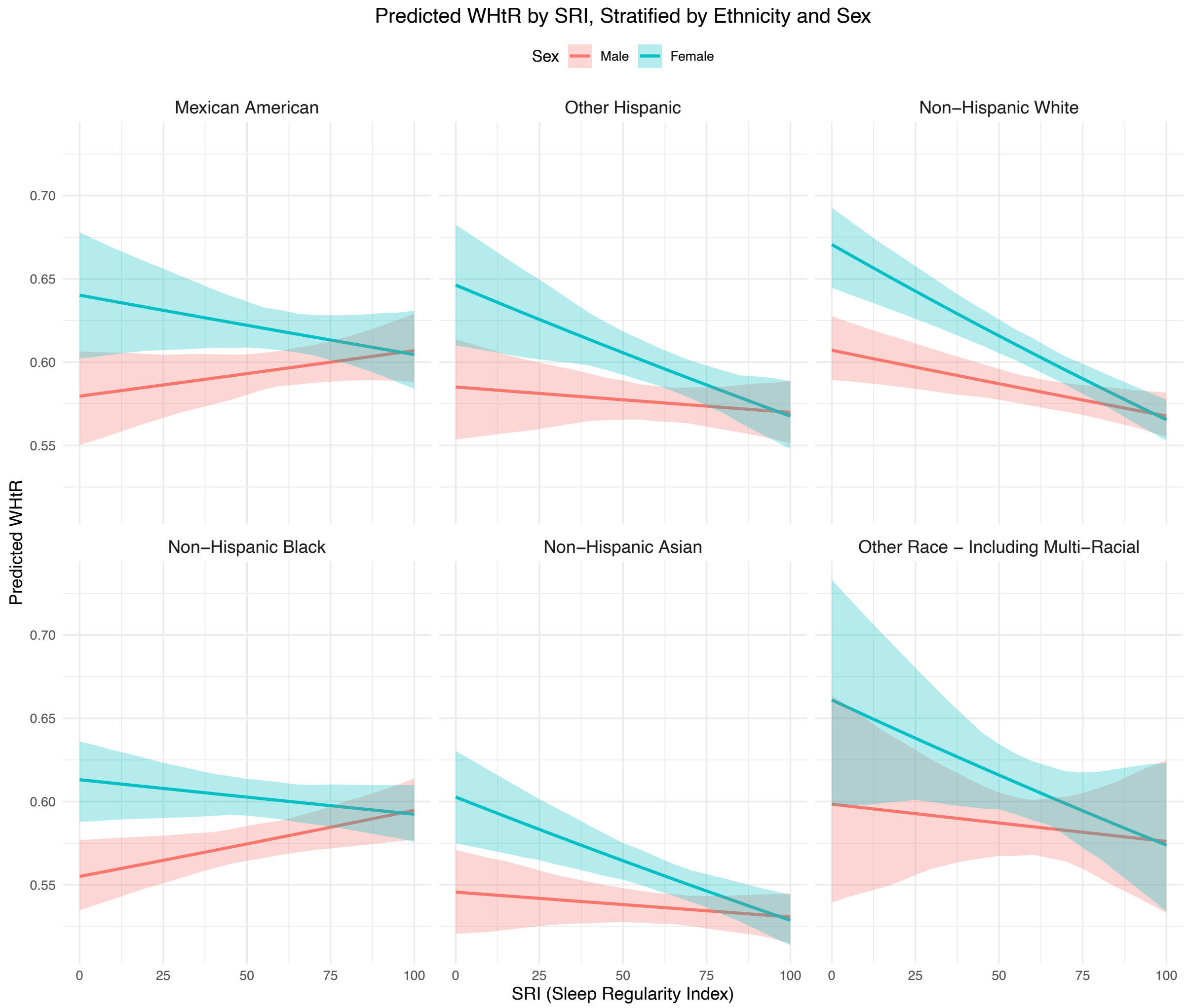
Predicted WHtR by SRI, Stratified by Ethnicity and Sex
Sex
Male
Female
Mexican American
Other Hispanic
Non−Hispanic White
Non−Hispanic Black
Non−Hispanic Asian
Other Race − Including Multi−Racial
Predicted WHtR
0.70
0.65
0.60
0.55
0
25
50
75
100
SRI (Sleep Regularity Index)

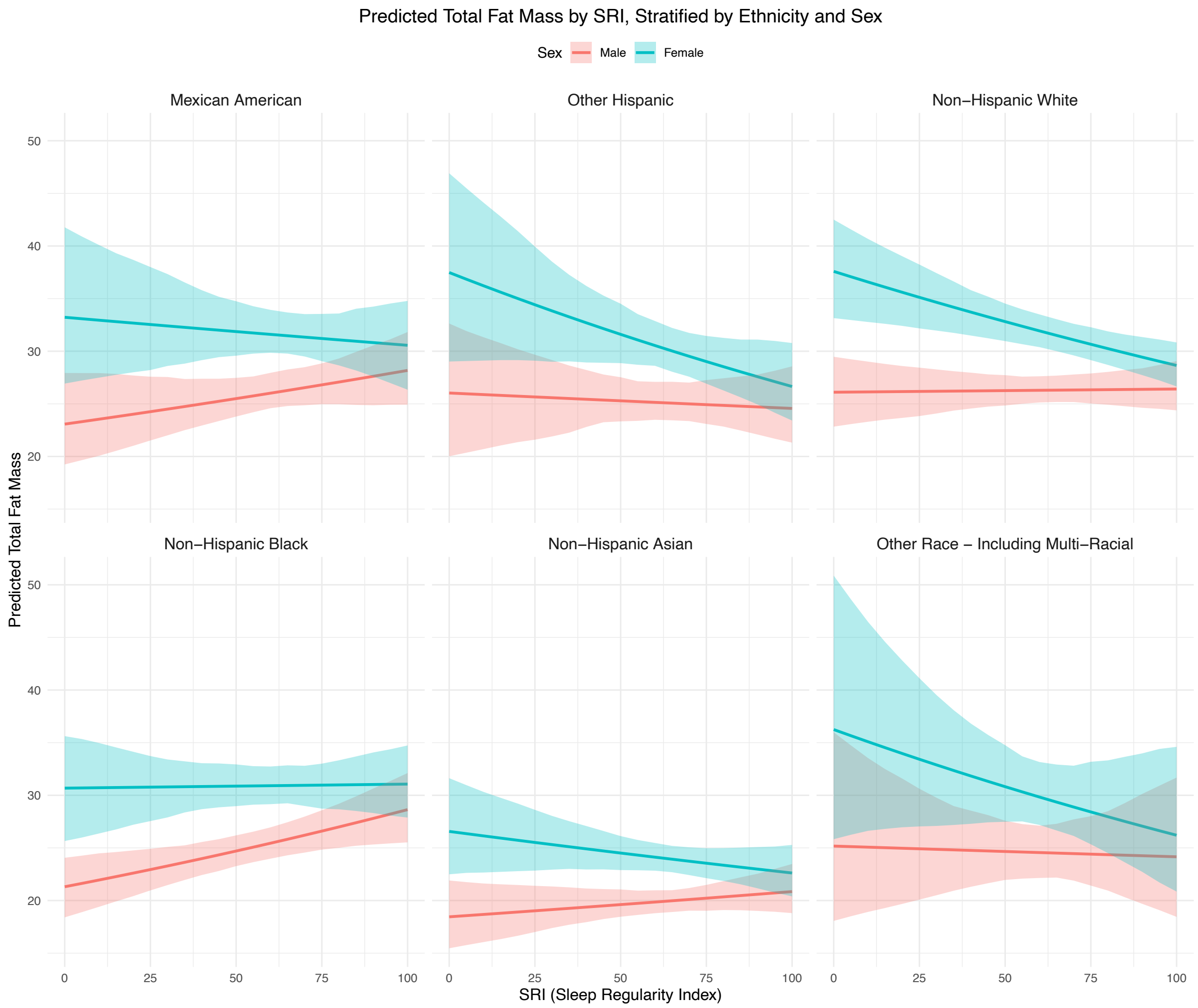

Predicted Total Fat Mass by SRI, Stratified by Ethnicity and Sex
Sex
Male
Female
Mexican American
Other Hispanic
Non−Hispanic White
Non−Hispanic Black
Non−Hispanic Asian
Other Race − Including Multi−Racial
Predicted Total Fat Mass
SRI (Sleep Regularity Index)
20
30
40
50
0
25
50
75
100

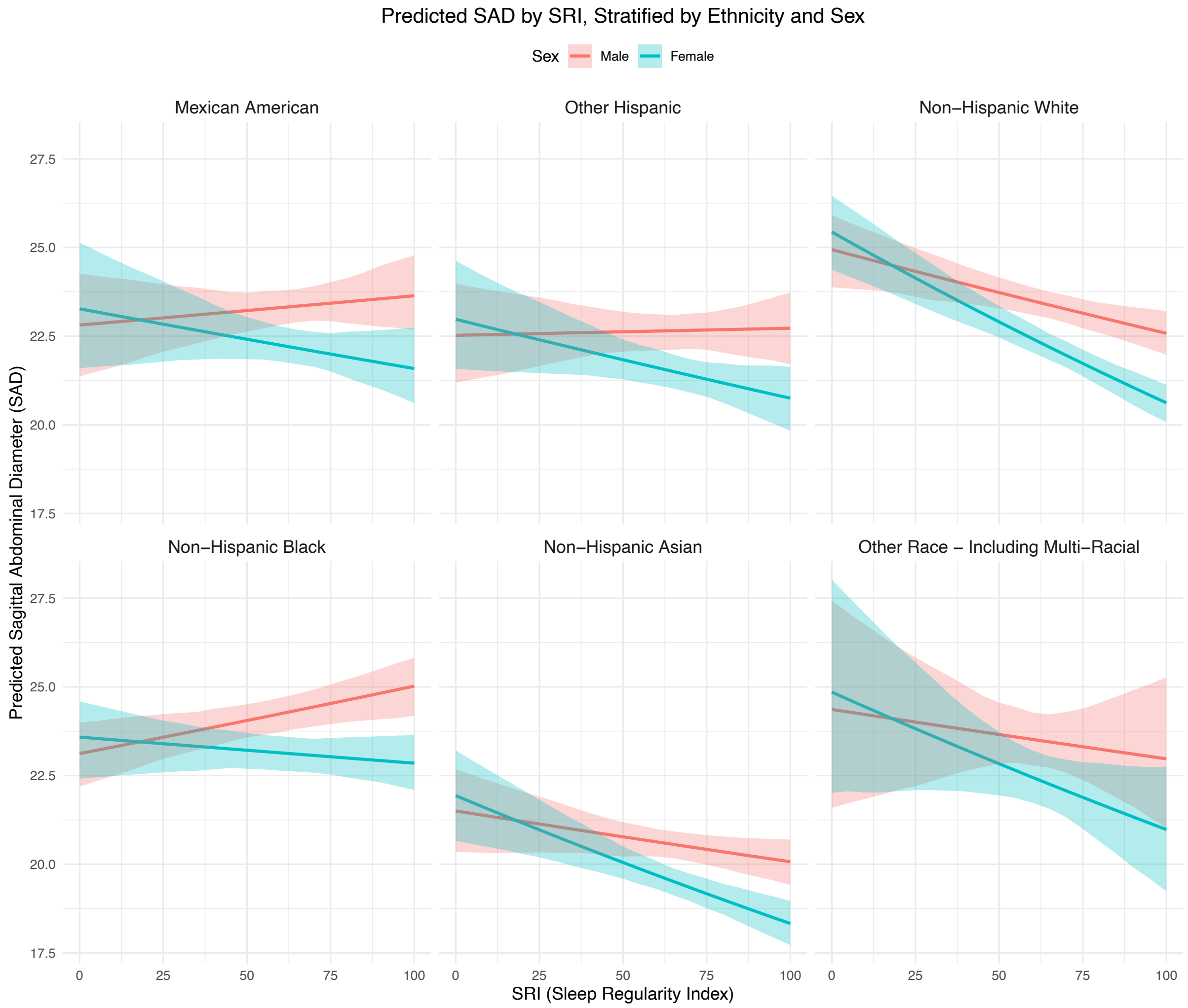

Predicted SAD by SRI, Stratified by Ethnicity and Sex
Sex
Male
Female
Mexican American
Other Hispanic
Non−Hispanic White
Non−Hispanic Black
Non−Hispanic Asian
Other Race − Including Multi−Racial
Predicted Sagittal Abdominal Diameter (SAD)
SRI (Sleep Regularity Index)
17.5
20.0
22.5
25.0
27.5
0
25
50
75
100

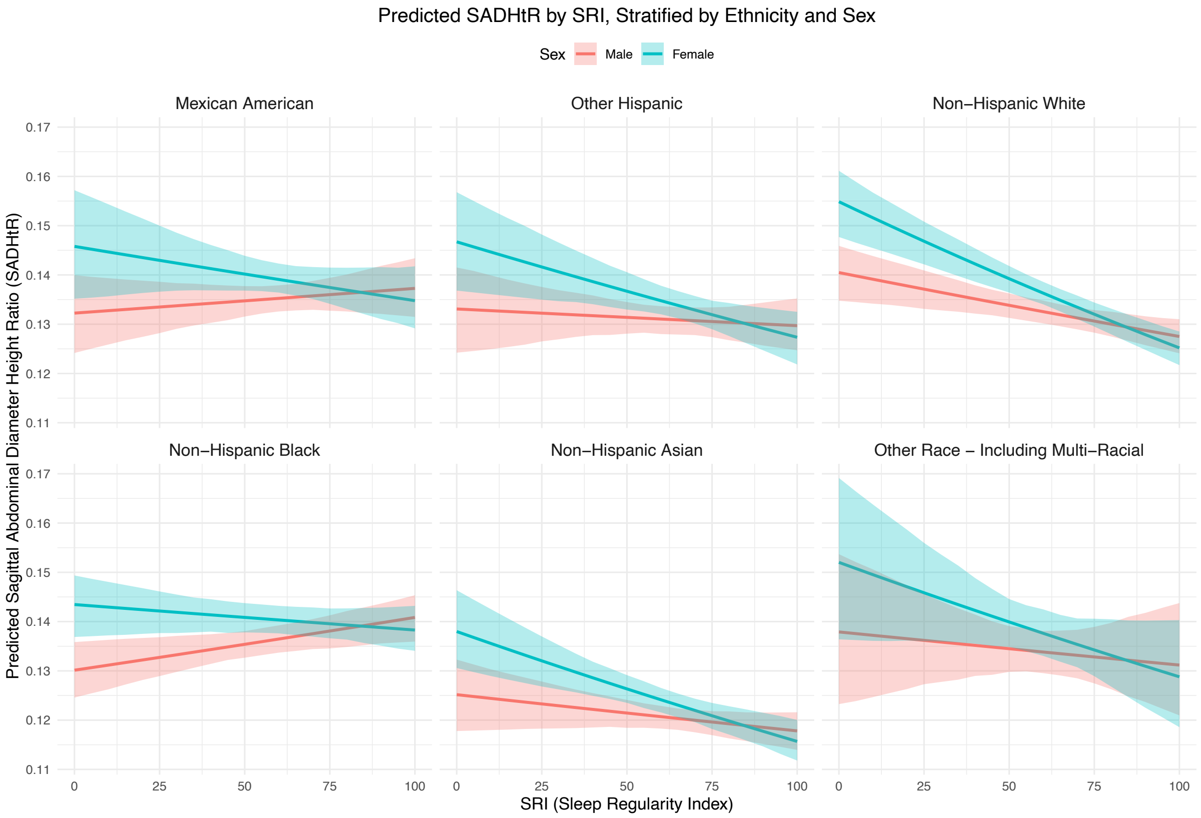

Predicted SADHtR by SRI, Stratified by Ethnicity and Sex
Sex
Male
Female
Mexican American
Other Hispanic
Non−Hispanic White
Non−Hispanic Black
Non−Hispanic Asian
Other Race − Including Multi−Racial
Predicted Sagittal Abdominal Diameter Height Ratio (SADHtR)
SRI (Sleep Regularity Index)
0.11
0.12
0.13
0.14
0.15
0.16
0.17
0
25
50
75
100

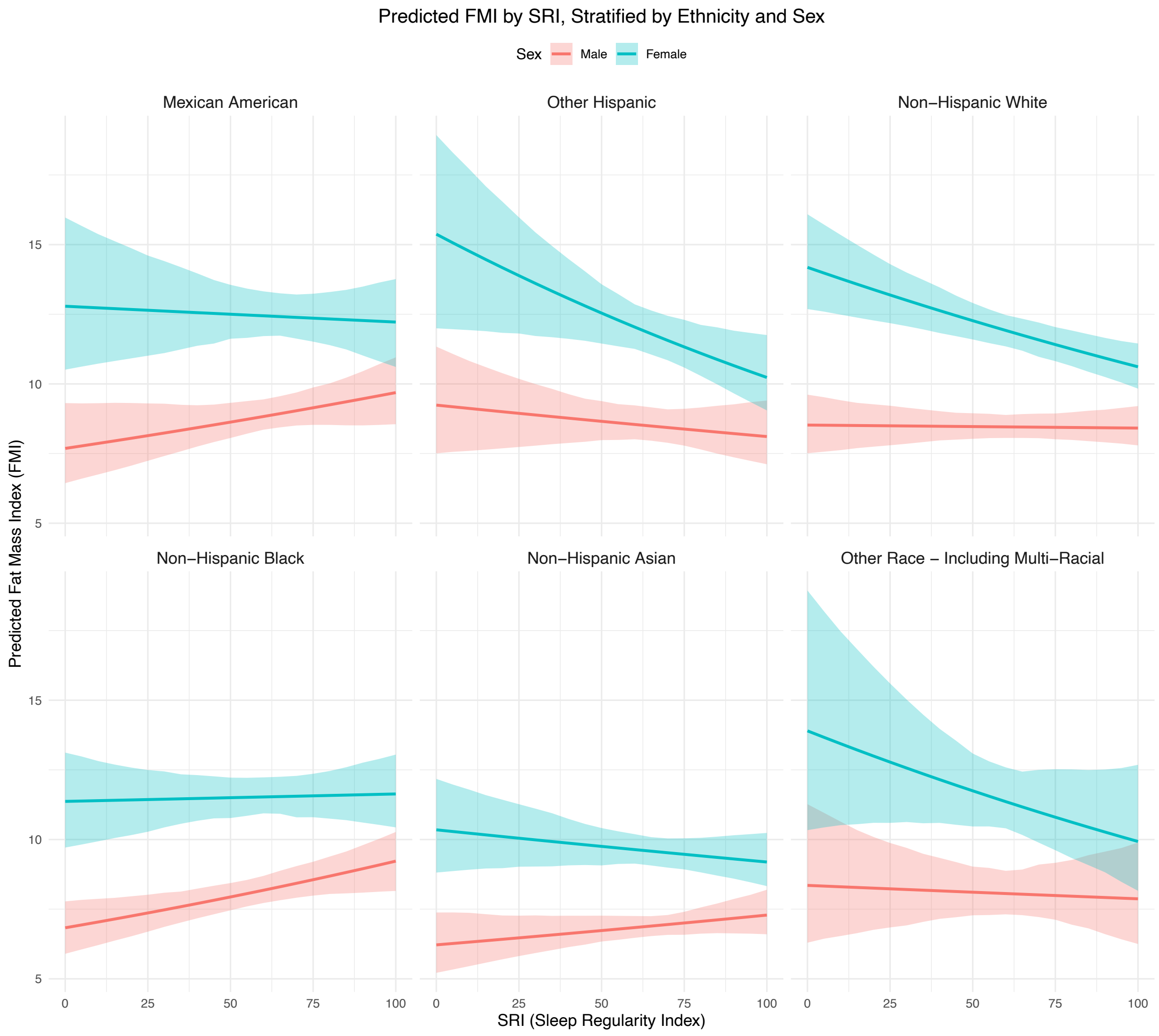

Predicted FMI by SRI, Stratified by Ethnicity and Sex
Sex
Male
Female
Mexican American
Other Hispanic
Non−Hispanic White
Non−Hispanic Black
Non−Hispanic Asian
Other Race − Including Multi−Racial
Predicted Fat Mass Index (FMI)
SRI (Sleep Regularity Index)
5
10
15
0
25
50
75
100

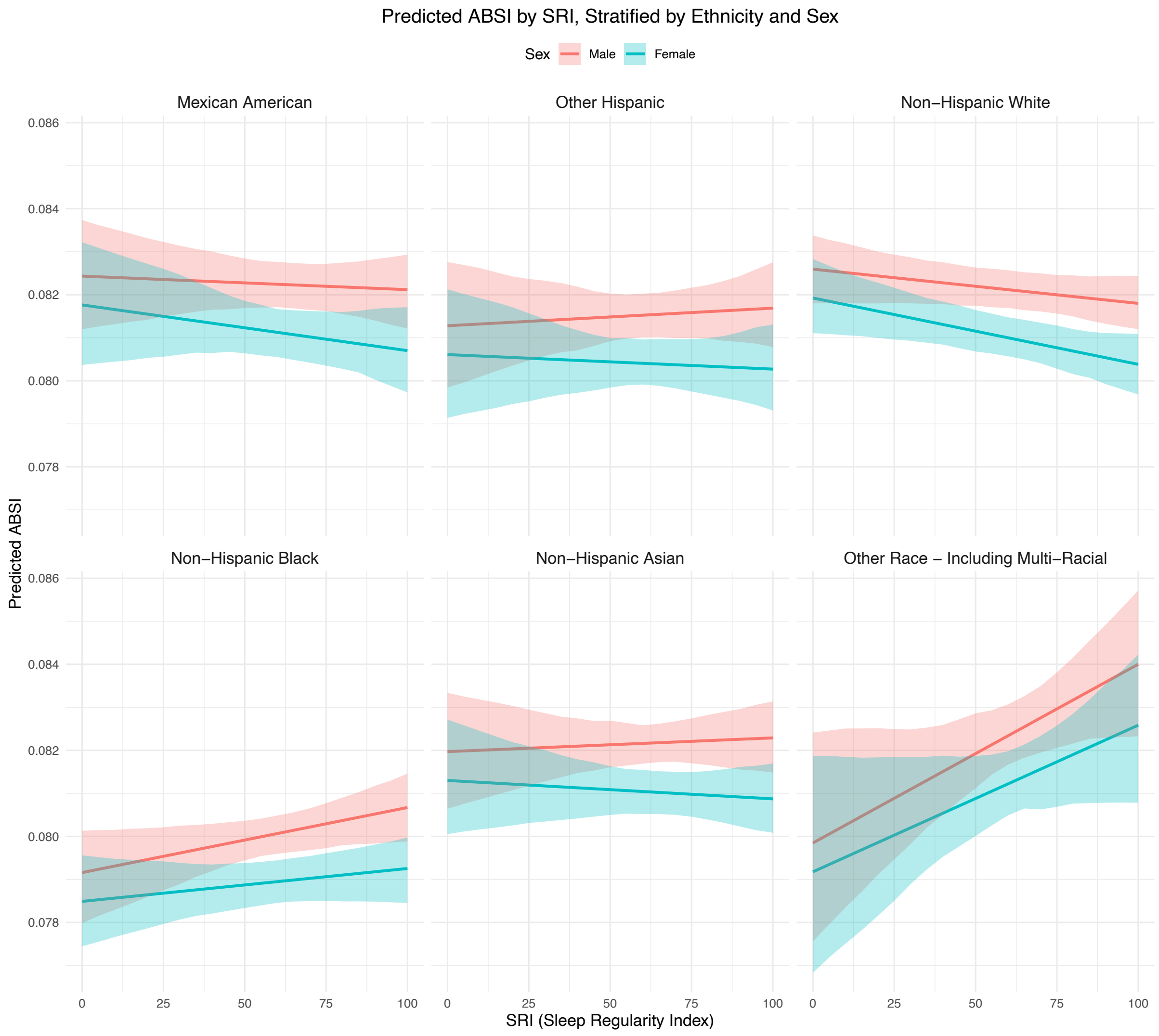

Predicted ABSI by SRI, Stratified by Ethnicity and Sex
Sex
Male
Female
Mexican American
Other Hispanic
Non−Hispanic White
Non−Hispanic Black
Non−Hispanic Asian
Other Race − Including Multi−Racial
Predicted ABSI
0.086
0.084
0.082
0.080
0.078
0
25
50
75
100
SRI (Sleep Regularity Index)

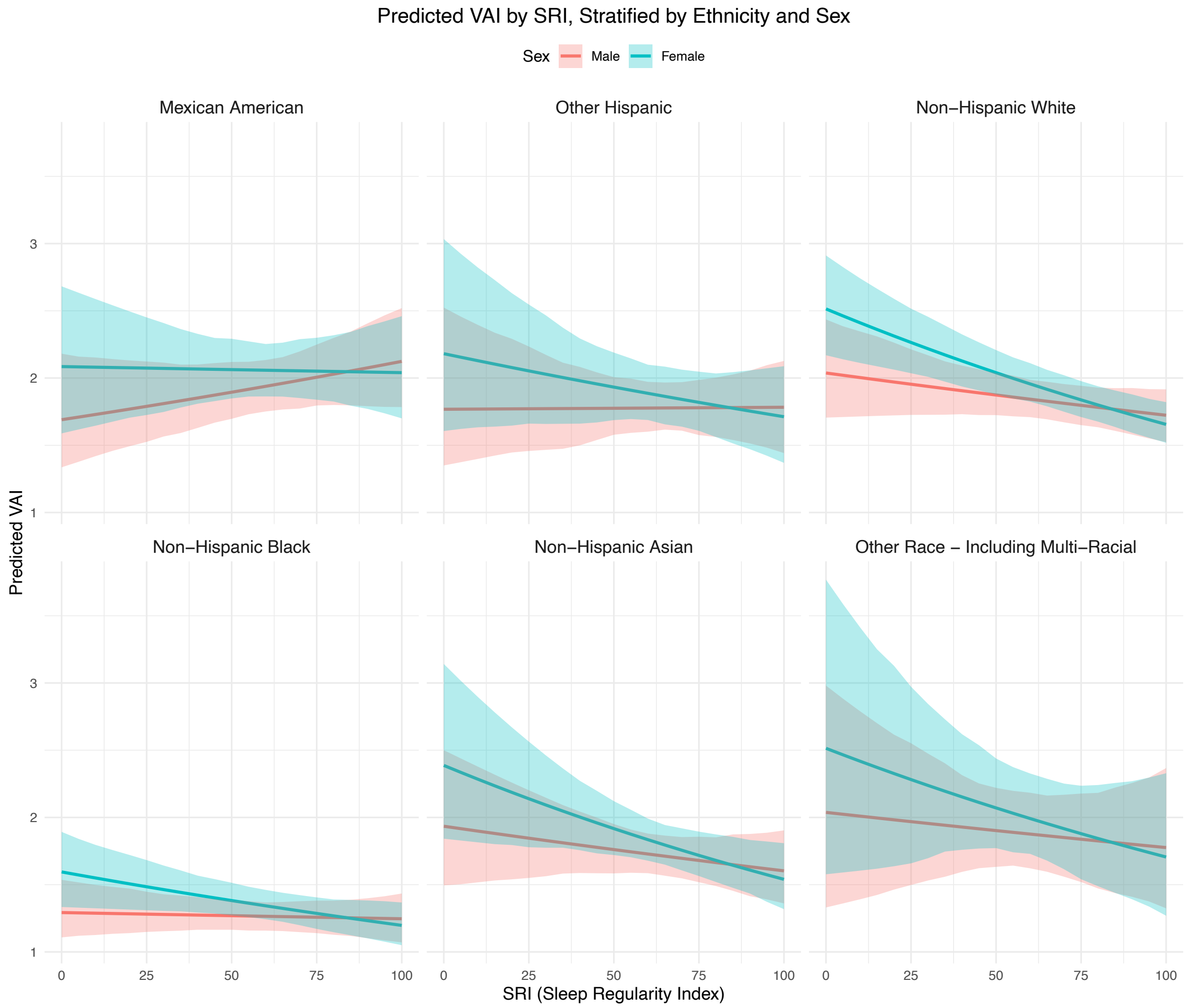

Predicted VAI by SRI, Stratified by Ethnicity and Sex
Sex
Male
Female
Mexican American
Other Hispanic
Non−Hispanic White
Non−Hispanic Black
Non−Hispanic Asian
Other Race − Including Multi−Racial
Predicted VAI
SRI (Sleep Regularity Index)
1
2
3
0
25
50
75
100

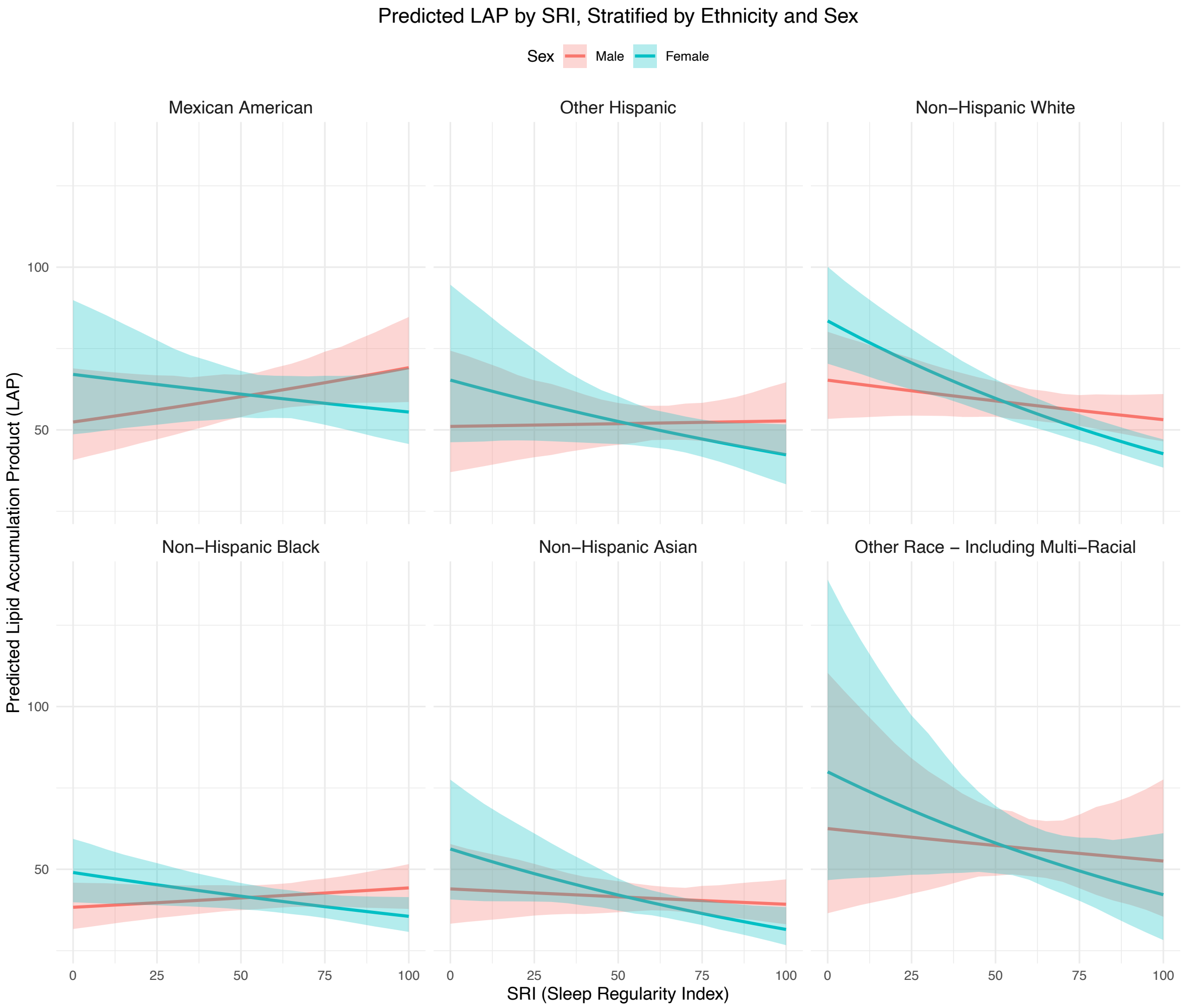
Predicted LAP by SRI, Stratified by Ethnicity and Sex
Sex
Male
Female
Mexican American
Other Hispanic
Non−Hispanic White
Non−Hispanic Black
Non−Hispanic Asian
Other Race − Including Multi−Racial
Predicted Lipid Accumulation Product (LAP)
100
50
0
25
50
75
100
SRI (Sleep Regularity Index)

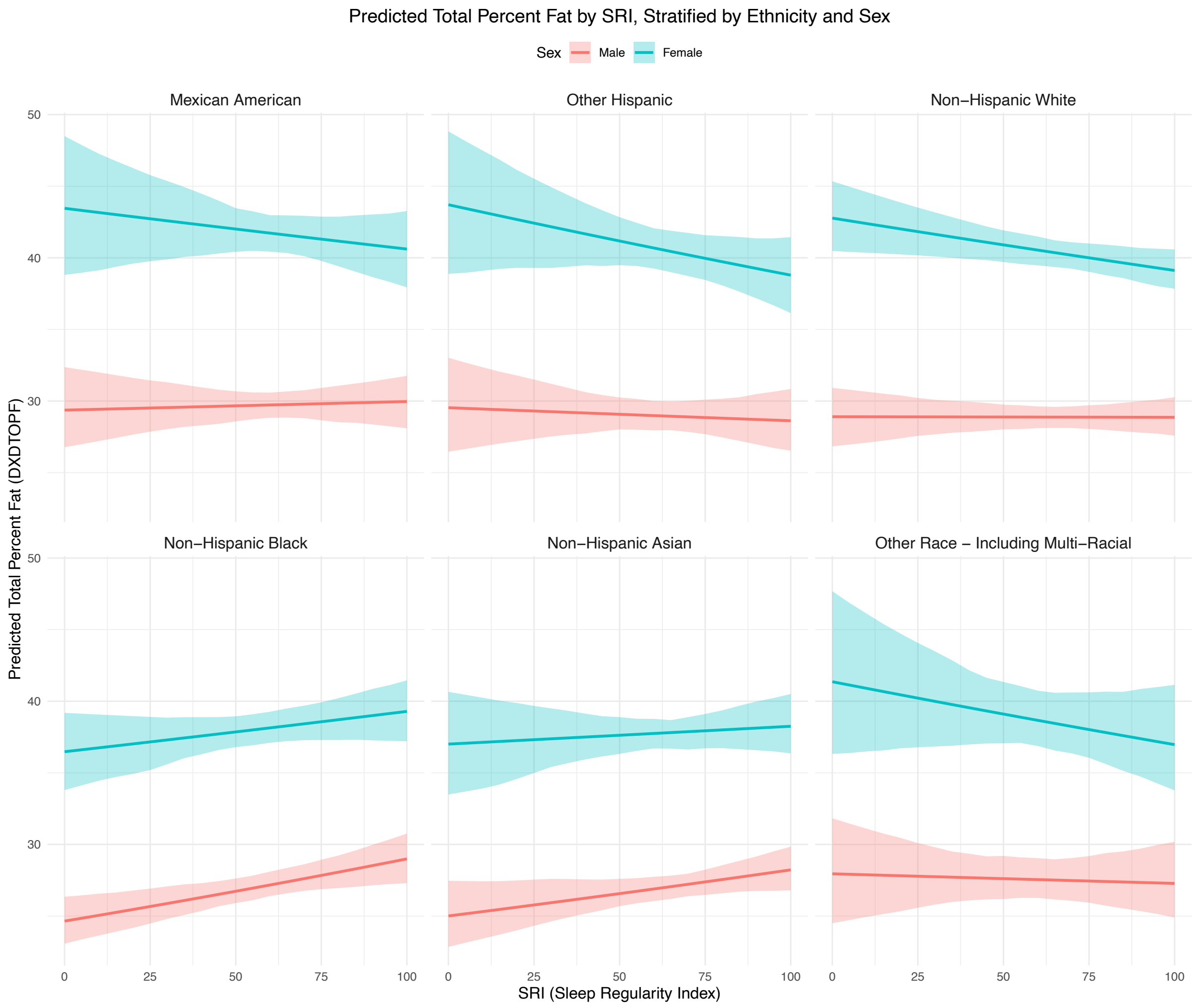

Predicted Total Percent Fat by SRI, Stratified by Ethnicity and Sex
Sex
Male
Female
Mexican American
Other Hispanic
Non−Hispanic White
Non−Hispanic Black
Non−Hispanic Asian
Other Race − Including Multi−Racial
Predicted Total Percent Fat (DXDTOPF)
SRI (Sleep Regularity Index)
50
40
30
0
25
50
75
100

**Table 1S.** Characteristics of the study population showing only participants from NHANES 2011-12 (without survey weights) stratified by quintiles of sleep regularity index (SRI).

| | **Overall** N = 3,677 | **SRI Q1** N = 736 | **SRI Q2** N = 735 | **SRI Q3** N = 735 | **SRI Q4** N = 735 | **SRI Q5** N = 736 |
|---|---|---|---|---|---|---|
| **Sleep Regularity Index (SRI)** [1] | 62 (49, 72) | 35 (27, 41) | 52 (49, 55) | 62 (59, 64) | 70 (68, 72) | 79 (76, 82) |
| **Age**[1] | 51 (36, 65) | 56 (36, 70) | 52 (36, 67) | 51 (35, 65) | 50 (37, 64) | 47 (36, 61) |
| **Gender**[2] | | | | | | |
| Male | 1,750 (48%) | 424 (58%) | 351 (48%) | 340 (46%) | 335 (46%) | 300 (41%) |
| Female | 1,927 (52%) | 312 (42%) | 384 (52%) | 395 (54%) | 400 (54%) | 436 (59%) |
| **Race/Ethnicity**[2] | | | | | | |
| Mexican American | 359 (9.8%) | 47 (6.4%) | 72 (9.8%) | 85 (12%) | 84 (11%) | 71 (9.6%) |
| Other Hispanic | 378 (10%) | 54 (7.3%) | 59 (8.0%) | 62 (8.4%) | 110 (15%) | 93 (13%) |
| Non-Hispanic White | 1,408 (38%) | 273 (37%) | 269 (37%) | 279 (38%) | 256 (35%) | 331 (45%) |
| Non-Hispanic Black | 974 (26%) | 272 (37%) | 232 (32%) | 199 (27%) | 163 (22%) | 108 (15%) |
| Non-Hispanic Asian | 451 (12%) | 70 (9.5%) | 78 (11%) | 89 (12%) | 103 (14%) | 111 (15%) |
| Other Race - Including Multi-Racial | 107 (2.9%) | 20 (2.7%) | 25 (3.4%) | 21 (2.9%) | 19 (2.6%) | 22 (3.0%) |
| **Education Level**[2] | | | | | | |
| Less Than 9th Grade | 354 (9.6%) | 86 (12%) | 77 (10%) | 63 (8.6%) | 72 (9.8%) | 56 (7.6%) |
| 9-11th Grade (Includes 12th grade with no diploma) | 513 (14%) | 131 (18%) | 118 (16%) | 84 (11%) | 97 (13%) | 83 (11%) |

| | Overall N = 3,677 | SRI Q1 N = 736 | SRI Q2 N = 735 | SRI Q3 N = 735 | SRI Q4 N = 735 | SRI Q5 N = 736 |
|---|---|---|---|---|---|---|
| High School Grad/GED or Equivalent | 777 (21%) | 161 (22%) | 161 (22%) | 170 (23%) | 140 (19%) | 145 (20%) |
| Some College or AA degree | 1,102 (30%) | 245 (33%) | 229 (31%) | 225 (31%) | 217 (30%) | 186 (25%) |
| College Graduate or above | 931 (25%) | 113 (15%) | 150 (20%) | 193 (26%) | 209 (28%) | 266 (36%) |
| **Household Income[2]** | | | | | | |
| < $20,000 | 908 (25%) | 274 (37%) | 207 (28%) | 183 (25%) | 132 (18%) | 112 (15%) |
| ≥ $20,000 | 2,769 (75%) | 462 (63%) | 528 (72%) | 552 (75%) | 603 (82%) | 624 (85%) |
| **Occupational Category[2]** | | | | | | |
| Working | 1,867 (51%) | 232 (32%) | 308 (42%) | 395 (54%) | 435 (59%) | 497 (68%) |
| Retired or Student | 880 (24%) | 244 (33%) | 197 (27%) | 172 (23%) | 151 (21%) | 116 (16%) |
| Unable to Work (Health or Family Reasons) | 622 (17%) | 182 (25%) | 149 (20%) | 105 (14%) | 108 (15%) | 78 (11%) |
| Unemployed | 210 (5.7%) | 52 (7.1%) | 54 (7.3%) | 41 (5.6%) | 28 (3.8%) | 35 (4.8%) |
| Other | 98 (2.7%) | 26 (3.5%) | 27 (3.7%) | 22 (3.0%) | 13 (1.8%) | 10 (1.4%) |
| **Marital Status[2]** | | | | | | |
| Married | 1,810 (49%) | 259 (35%) | 336 (46%) | 356 (48%) | 397 (54%) | 462 (63%) |
| Widowed | 341 (9.3%) | 104 (14%) | 79 (11%) | 64 (8.7%) | 55 (7.5%) | 39 (5.3%) |

| | **Overall** N = 3,677 | **SRI Q1** N = 736 | **SRI Q2** N = 735 | **SRI Q3** N = 735 | **SRI Q4** N = 735 | **SRI Q5** N = 736 |
|---|---|---|---|---|---|---|
| Divorced | 410 (11%) | 97 (13%) | 83 (11%) | 85 (12%) | 76 (10%) | 69 (9.4%) |
| Separated | 130 (3.5%) | 36 (4.9%) | 23 (3.1%) | 18 (2.4%) | 30 (4.1%) | 23 (3.1%) |
| Never married | 712 (19%) | 189 (26%) | 157 (21%) | 149 (20%) | 125 (17%) | 92 (13%) |
| Living with partner | 274 (7.5%) | 51 (6.9%) | 57 (7.8%) | 63 (8.6%) | 52 (7.1%) | 51 (6.9%) |
| **Alcohol Consumption**[2] | | | | | | |
| Heavy Drinker | 109 (3.0%) | 29 (3.9%) | 30 (4.1%) | 21 (2.9%) | 16 (2.2%) | 13 (1.8%) |
| Moderate Drinker | 2,924 (80%) | 567 (77%) | 573 (78%) | 602 (82%) | 581 (79%) | 601 (82%) |
| Never/Non-Drinker | 644 (18%) | 140 (19%) | 132 (18%) | 112 (15%) | 138 (19%) | 122 (17%) |
| **Smoking Status**[2] | | | | | | |
| Heavy smoker | 413 (11%) | 130 (18%) | 91 (12%) | 76 (10%) | 65 (8.8%) | 51 (6.9%) |
| Light smoker | 223 (6.1%) | 76 (10%) | 52 (7.1%) | 45 (6.1%) | 31 (4.2%) | 19 (2.6%) |
| Moderate smoker | 79 (2.1%) | 18 (2.4%) | 22 (3.0%) | 15 (2.0%) | 12 (1.6%) | 12 (1.6%) |
| Nonsmokers | 2,070 (56%) | 341 (46%) | 394 (54%) | 391 (53%) | 464 (63%) | 480 (65%) |
| Previous smoker | 892 (24%) | 171 (23%) | 176 (24%) | 208 (28%) | 163 (22%) | 174 (24%) |
| **Vitamin D Level**[1] | 62 (45, 81) | 57 (38, 78) | 59 (41, 79) | 62 (44, 82) | 63 (47, 81) | 68 (52, 85) |

| | Overall<br>N = 3,677 | SRI Q1<br>N = 736 | SRI Q2<br>N = 735 | SRI Q3<br>N = 735 | SRI Q4<br>N = 735 | SRI Q5<br>N = 736 |
|---|---|---|---|---|---|---|
| **Total Caloric Intake**[1] | 1,924 (1,458, 2,568) | 1,851 (1,366, 2,552) | 1,890 (1,379, 2,501) | 1,952 (1,510, 2,574) | 1,954 (1,469, 2,576) | 1,993 (1,541, 2,603) |
| **Depression Score (PHQ-9)** [1] | 1.0 (0.0, 4.0) | 3.0 (0.0, 7.0) | 2.0 (0.0, 5.0) | 1.0 (0.0, 4.0) | 1.0 (0.0, 3.0) | 1.0 (0.0, 3.0) |
| **Activity Level**[1] | 6.7 (4.1, 9.8) | 4.3 (2.3, 7.0) | 5.7 (3.3, 8.6) | 6.8 (4.5, 9.5) | 8.0 (5.3, 10.9) | 8.9 (6.0, 12.2) |

[1]Median (Q1, Q3)

[2]n (%)

**Table 2S.** Characteristics of the study population showing only participants from NHANES 2013-14 (without survey weights) stratified by quintiles of sleep regularity index (SRI).

| | Overall<br>N = 3,408 | SRI Q1<br>N = 682 | SRI Q2<br>N = 681 | SRI Q3<br>N = 682 | SRI Q4<br>N = 681 | SRI Q5<br>N = 682 |
|---|---|---|---|---|---|---|
| **Sleep Regularity Index (SRI)** [1] | 61 (49, 71) | 36 (29, 42) | 52 (49, 55) | 61 (59, 63) | 69 (67, 71) | 78 (75, 81) |
| **Age**[1] | 52 (38, 66) | 58 (43, 71) | 55 (38, 69) | 51 (35, 65) | 50 (37, 63) | 47 (38, 60) |
| **Gender** | | | | | | |
| Male | 1,583 (46%) | 356 (52%) | 322 (47%) | 303 (44%) | 309 (45%) | 293 (43%) |
| Female | 1,825 (54%) | 326 (48%) | 359 (53%) | 379 (56%) | 372 (55%) | 389 (57%) |
| **Race/Ethnicity** | | | | | | |

| | Overall<br>N = 3,408 | SRI Q1<br>N = 682 | SRI Q2<br>N = 681 | SRI Q3<br>N = 682 | SRI Q4<br>N = 681 | SRI Q5<br>N = 682 |
|---|---|---|---|---|---|---|
| Mexican American | 467 (14%) | 60 (8.8%) | 77 (11%) | 100 (15%) | 122 (18%) | 108 (16%) |
| Other Hispanic | 304 (8.9%) | 40 (5.9%) | 59 (8.7%) | 67 (9.8%) | 72 (11%) | 66 (9.7%) |
| Non-Hispanic White | 1,500 (44%) | 316 (46%) | 290 (43%) | 283 (41%) | 279 (41%) | 332 (49%) |
| Non-Hispanic Black | 685 (20%) | 188 (28%) | 167 (25%) | 145 (21%) | 113 (17%) | 72 (11%) |
| Non-Hispanic Asian | 346 (10%) | 58 (8.5%) | 59 (8.7%) | 65 (9.5%) | 80 (12%) | 84 (12%) |
| Other Race - Including Multi-Racial | 106 (3.1%) | 20 (2.9%) | 29 (4.3%) | 22 (3.2%) | 15 (2.2%) | 20 (2.9%) |
| **Education Level** | | | | | | |
| Less Than 9th Grade | 289 (8.5%) | 53 (7.8%) | 48 (7.0%) | 58 (8.5%) | 78 (11%) | 52 (7.6%) |
| 9-11th Grade (Includes 12th grade with no diploma) | 459 (13%) | 108 (16%) | 93 (14%) | 91 (13%) | 81 (12%) | 86 (13%) |
| High School Grad/GED or Equivalent | 789 (23%) | 172 (25%) | 160 (23%) | 173 (25%) | 150 (22%) | 134 (20%) |
| Some College or AA degree | 1,023 (30%) | 215 (32%) | 236 (35%) | 192 (28%) | 196 (29%) | 184 (27%) |
| College Graduate or above | 848 (25%) | 134 (20%) | 144 (21%) | 168 (25%) | 176 (26%) | 226 (33%) |
| **Household Income** | | | | | | |
| < $20,000 | 721 (21%) | 209 (31%) | 164 (24%) | 136 (20%) | 122 (18%) | 90 (13%) |

| | Overall N = 3,408 | SRI Q1 N = 682 | SRI Q2 N = 681 | SRI Q3 N = 682 | SRI Q4 N = 681 | SRI Q5 N = 682 |
|---|---|---|---|---|---|---|
| ≥ $20,000 | 2,687 (79%) | 473 (69%) | 517 (76%) | 546 (80%) | 559 (82%) | 592 (87%) |
| **Occupational Category** | | | | | | |
| Working | 1,779 (52%) | 219 (32%) | 306 (45%) | 361 (53%) | 412 (60%) | 481 (71%) |
| Retired or Student | 803 (24%) | 228 (33%) | 202 (30%) | 146 (21%) | 130 (19%) | 97 (14%) |
| Unable to Work (Health or Family Reasons) | 640 (19%) | 189 (28%) | 129 (19%) | 134 (20%) | 104 (15%) | 84 (12%) |
| Unemployed | 116 (3.4%) | 28 (4.1%) | 25 (3.7%) | 27 (4.0%) | 24 (3.5%) | 12 (1.8%) |
| Other | 70 (2.1%) | 18 (2.6%) | 19 (2.8%) | 14 (2.1%) | 11 (1.6%) | 8 (1.2%) |
| **Marital Status** | | | | | | |
| Married | 1,798 (53%) | 271 (40%) | 334 (49%) | 362 (53%) | 385 (57%) | 446 (65%) |
| Widowed | 301 (8.8%) | 89 (13%) | 65 (9.5%) | 55 (8.1%) | 54 (7.9%) | 38 (5.6%) |
| Divorced | 414 (12%) | 108 (16%) | 93 (14%) | 76 (11%) | 85 (12%) | 52 (7.6%) |
| Separated | 105 (3.1%) | 32 (4.7%) | 21 (3.1%) | 20 (2.9%) | 21 (3.1%) | 11 (1.6%) |
| Never married | 585 (17%) | 148 (22%) | 137 (20%) | 115 (17%) | 94 (14%) | 91 (13%) |
| Living with partner | 205 (6.0%) | 34 (5.0%) | 31 (4.6%) | 54 (7.9%) | 42 (6.2%) | 44 (6.5%) |
| **Alcohol Consumption** | | | | | | |

| | **Overall** N = 3,408 | **SRI Q1** N = 682 | **SRI Q2** N = 681 | **SRI Q3** N = 682 | **SRI Q4** N = 681 | **SRI Q5** N = 682 |
|---|---|---|---|---|---|---|
| Heavy Drinker | 67 (2.0%) | 15 (2.2%) | 12 (1.8%) | 17 (2.5%) | 14 (2.1%) | 9 (1.3%) |
| Moderate Drinker | 2,761 (81%) | 548 (80%) | 557 (82%) | 556 (82%) | 536 (79%) | 564 (83%) |
| Never/Non-Drinker | 580 (17%) | 119 (17%) | 112 (16%) | 109 (16%) | 131 (19%) | 109 (16%) |
| **Smoking Status** | | | | | | |
| Heavy smoker | 356 (10%) | 102 (15%) | 83 (12%) | 70 (10%) | 54 (7.9%) | 47 (6.9%) |
| Light smoker | 229 (6.7%) | 67 (9.8%) | 53 (7.8%) | 39 (5.7%) | 41 (6.0%) | 29 (4.3%) |
| Moderate smoker | 58 (1.7%) | 14 (2.1%) | 17 (2.5%) | 5 (0.7%) | 15 (2.2%) | 7 (1.0%) |
| Nonsmokers | 1,906 (56%) | 323 (47%) | 342 (50%) | 398 (58%) | 405 (59%) | 438 (64%) |
| Previous smoker | 859 (25%) | 176 (26%) | 186 (27%) | 170 (25%) | 166 (24%) | 161 (24%) |
| **Vitamin D Level**[1] | 64 (48, 83) | 61 (42, 82) | 64 (46, 83) | 64 (49, 84) | 65 (50, 82) | 67 (54, 84) |
| **Total Caloric Intake**[1] | 1,906 (1,425, 2,521) | 1,789 (1,300, 2,396) | 1,831 (1,367, 2,441) | 1,945 (1,427, 2,575) | 1,936 (1,478, 2,543) | 2,001 (1,546, 2,595) |
| **Depression Score (PHQ-9)** [1] | 2.0 (0.0, 5.0) | 3.0 (1.0, 7.0) | 2.0 (0.0, 5.0) | 2.0 (0.0, 5.0) | 1.0 (0.0, 4.0) | 1.0 (0.0, 3.0) |
| **Activity Level**[1] | 8.7 (6.3, 11.8) | 5.7 (4.0, 8.2) | 7.4 (5.6, 10.3) | 8.9 (6.8, 11.5) | 10.3 (7.7, 12.8) | 11.1 (8.6, 13.8) |

[1]Median (Q1, Q3)

[2]n (%)

## References


CDC. *Physical Activity Monitor - Minute (PAXMIN_G)*. Retrieved 15.8. from https://wwwn.cdc.gov/Nchs/Data/Nhanes/Public/2011/DataFiles/BMX_G.htm

CDC. *Physical Activity Monitor - Minute (PAXMIN_H)*. Retrieved 15.8. from https://wwwn.cdc.gov/Nchs/Data/Nhanes/Public/2013/DataFiles/BMX_H.htm

Herrick, K. A., Storandt, R. J., Afful, J., Pfeiffer, C. M., Schleicher, R. L., Gahche, J. J., & Potischman, N. (2019). Vitamin D status in the United States, 2011-2014. *Am J Clin Nutr*, *110*(1), 150-157. https://doi.org/10.1093/ajcn/nqz037

Kowarik, A., & Templ, M. (2016). Imputation with the R Package VIM. *Journal of Statistical Software*, *74*(7), 1 - 16. https://doi.org/10.18637/jss.v074.i07

Kroenke, K., & Spitzer, R. L. (2002). The PHQ-9: a new depression diagnostic and severity measure. In (Vol. 32, pp. 509-515): Slack Incorporated Thorofare, NJ.

Tierney, N. (2017). visdat: Visualising Whole Data Frames. *JOSS*, *2*(16), 355. https://doi.org/10.21105/joss.00355

Westfall, P. H., & Arias, A. L. (2020). *Understanding regression analysis: a conditional distribution approach*. Chapman and Hall/CRC.
